# Supplementary figures and images for: Soil-transmitted helminth infections and nutritional status in Ecuador: findings from a national survey and implications for control strategies
Source: BMJ Open. 2018 Apr 28;8(4):e021319. doi: 10.1136/bmjopen-2017-021319 (PMC5931300; doi:10.1136/bmjopen-2017-021319)

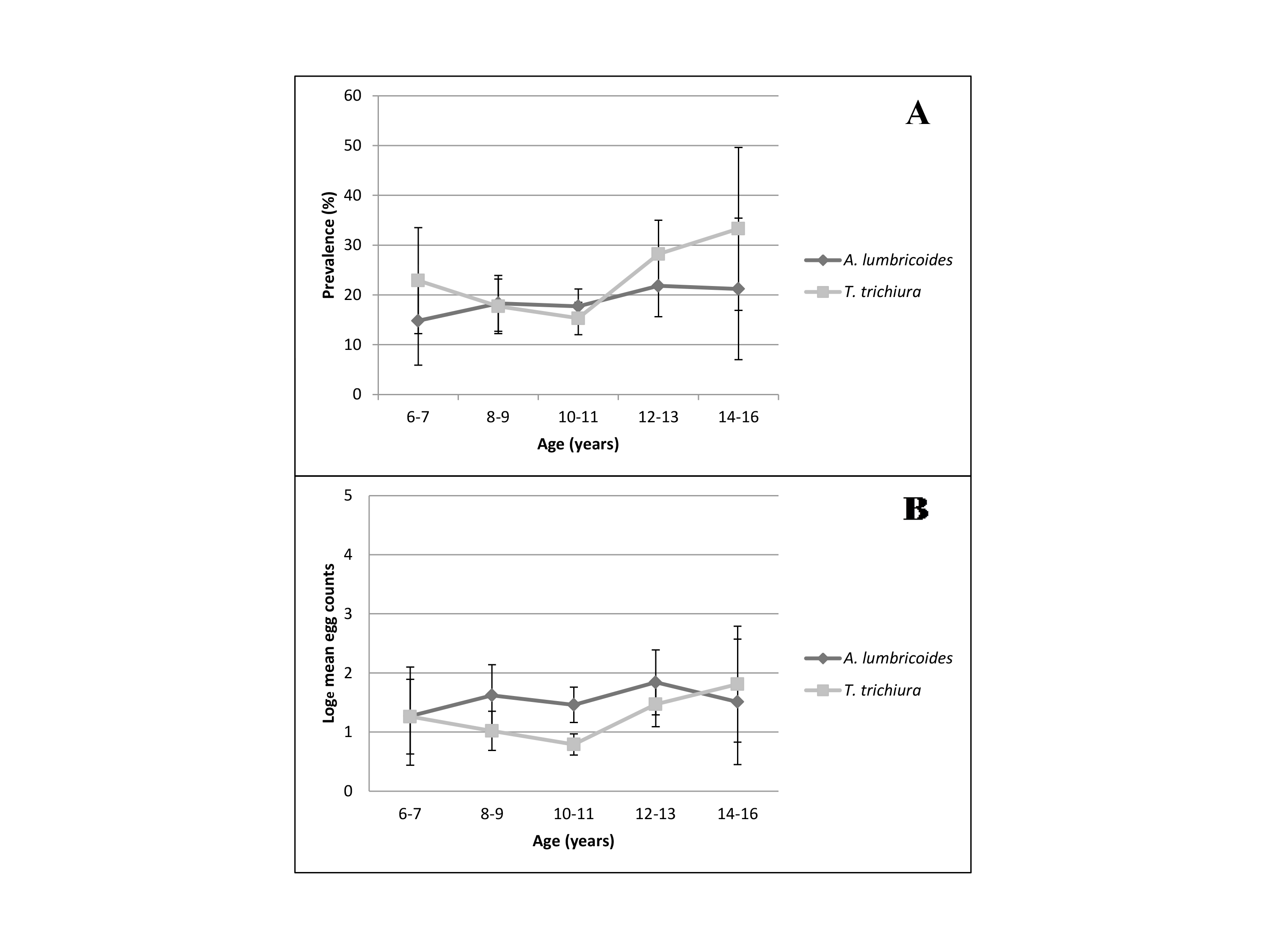

Supplement: Supplementary file 1 [file bmjopen-2017-021319supp001.jpg]
