# Supplementary material for: Soil-transmitted helminth infections and nutritional status in Ecuador: findings from a national survey and implications for control strategies
Source: BMJ Open. 2018 Apr 28;8(4):e021319. doi: 10.1136/bmjopen-2017-021319 (PMC5931300; doi:10.1136/bmjopen-2017-021319)
Supplement: Author's manuscript [file bmjopen-2017-021319.draft_revisions.pdf]

BMJ Open

BMJ Open is committed to open peer review. As part of this commitment we make the peer review history of every article we publish publicly available.

When an article is published we post the peer reviewers' comments and the authors' responses online. We also post the versions of the paper that were used during peer review. These are the versions that the peer review comments apply to.

The versions of the paper that follow are the versions that were submitted during the peer review process. They are not the versions of record or the final published versions. They should not be cited or distributed as the published version of this manuscript.

BMJ Open is an open access journal and the full, final, typeset and author-corrected version of record of the manuscript is available on our site with no access controls, subscription charges or pay-per-view fees (http://bmjopen.bmj.com).

If you have any questions on BMJ Open's open peer review process please email info.bmjopen@bmj.com

BMJ Open

Soil-transmitted helminth infections and nutritional status in Ecuador: findings from a national survey and implications for control strategies

Journal:	*BMJ Open*
Manuscript ID	bmjopen-2017-021319
Article Type:	Research
Date Submitted by the Author:	22-Dec-2017
Complete List of Authors:	Moncayo, Ana-Lucia; Pontificia Universidad Catolica del Ecuador<br>Lovato, Raquel; Ministerio de Salud Publica<br>Cooper, Philip; St George's University of London, Institute of Infection and Immunity
Keywords:	soil-transmitted helminths, national survey, Ecuador, malnutrition

SCHOLARONE™
Manuscripts

For peer review only

Soil-transmitted helminth infections and nutritional status in Ecuador: findings from a national survey and implications for control strategies

Moncayo AL[1], Lovato R[2], Cooper PJ[3,4]

[1]Centro de Investigación Para la Salud en América Latina (CISeAL), Pontificia Universidad Católica del Ecuador, Quito, Ecuador
[2]Dirección de Vigilancia Epidemiológica, Ministerio de Salud Pública del Ecuador, Quito ,Ecuador
[3] Facultad de Ciencias Médicas, de la Salud y la Vida, Universidad Internacional del Ecuador, Quito, Ecuador .
[4]Institute of Infection and Immunity, St George's University of London, UK.

Correspondence to: amoncayo708@puce.edu.ec

For peer review only

Abstract

Objective The estimation of prevalence and intensity of soil-transmitted helminth infections (STH) at a country-level is an essential pre-requisite for the implementation of a rational control program. The aim of the present study was to estimate the prevalence and distribution of STH infections and malnutrition in school age children in rural areas of Ecuador.

Design Cross-sectional study from October 2011 and May 2012.

Setting Eighteen rural schools were randomly selected from the three ecological regions of Ecuador (coastal, highlands and Amazon basin).

Participants 920 children aged 6-16 years old.

Main outcome measures prevalence and intensity of STH infections associated with malnutrition (thinness/wasting or stunting)

Results: The results showed that 257 (27.9%) children were infected with at least one STH parasite. The prevalence of *Trichuris trichiura*, *Ascaris lumbricoides* and hookworm was 19.3%, 18.5%, and 5.0%, respectively. Malnutrition was present in 14.1 % of children and most common was stunting (12.3%). Compared to other regions, schoolchildren in the Amazon region had the highest STH prevalence (58.9%) of which a greater proportion of infections were moderate/heavy intensity (45.6%) and had the highest prevalence of malnutrition (14.1%). A positive association was observed between heavy infections with *A. lumbricoides* and malnutrition (OR 3.70, 95% CI 1.48 to 9.24, $p = 0.005$).

For peer review only

Conclusions: Our estimate of the prevalence of STH infections of 27.9% at a national level in Ecuador is lower than suggested by previous studies. Our data indicate that schoolchildren living in the Amazon region have a greater risk of STH infection and stunting compared with children from other regions. The implementation of control programs within this region, therefore, is a priority and should include both deworming and health education strategies.

For peer review only

Strengths and limitations of this study

- These data are from the first national survey of STH infections in schoolchildren in Ecuador and will inform the strategy for a national plan for deworming of schoolchildren in the three ecological regions in Ecuador.
- These baseline data will allow health authorities to monitor the impact of future deworming programmes among schoolchildren.
- The sampling strategy may have not detected localized geographic areas within the three regions with a higher prevalence of STH infections.
- The cross-sectional nature of the study does not allow us to determine the temporal relationship between the STH infections and malnutrition.
- A single stool sample may underestimate STH prevalence.

Introduction

Soil-transmitted helminth infections (STH) (*Ascaris lumbricoides*, *Trichuris trichiura* and hookworm) are a major public health problem in tropical and sub-tropical regions of developing countries, especially in marginalized population with poor access to clean water and sanitation, and living in overcrowded conditions with low levels of education and lack of access to health services.[1] Recent estimates suggest that more than 1.45 billion humans worldwide are infected with STH parasites causing up to 4.98 million Years Lost due to Disability and 5.18 Disability-Adjusted Life Years.[2] Children are the group at highest risk of infection and most vulnerable to the pathologic consequences of infection. An estimated 13.9 million preschool and 35.4 million schoolchildren in Latin America and the Caribbean (LAC) were at risk of infection by STH in 2012.[3] STH infections are considered to have important deleterious effects on the nutritional status, growth, and physical development of infected children,[4 5] and may also affect cognitive performance and educability.[5]

In the last decade, an increasing number of international initiatives have established the aim to either reduce or to eliminate the disease burden caused by STHs and other helminth parasites prevalent in resource-poor regions. Chemotherapy still remains the most effective short-term approach for STH control in areas where infections are highly endemic. The World Health Assembly in 2001 urged all Member States where STH infections are endemic to treat at least 75% and up to 100% of all school-age children at risk of morbidity by 2010.[6] In Ecuador, international agencies and non-governmental organizations (NGOs) have formed partnerships with the government to provide deworming treatments for a large number of schoolchildren, and achieved the goal of at least 75% coverage of eligible

For peer review only

schoolchildren in 2006 and 2009, years for which data are available.[7] However, since 2009, there are no available data on treatment coverage of preschool and school-age children, indicating a lack of progress in the implementation of this important public health intervention.

Few countries in LAC have implemented nationwide surveys on prevalence and intensity of STH infections in order to plan treatment strategies. A recent study identified gaps in available data on STH infections using data published between 2000 and 2010 in LAC.[8] A total of 335 published studies of STH prevalence were found in 18 countries: in Ecuador, 11 studies were analyzed of which 2 estimated a prevalence below 20%, 4 (36.4%) estimated a prevalence between 20-50% and 5 (45.5%) a prevalence of >50%. These data come from highly focal studies restricted to one or more parishes in 7 of the country's 24 provinces.

The aim of the present study was to carry out a national survey to estimate the current prevalence and intensity of STH infections and malnutrition in school age children. These data will be use to design and evaluate appropriate intervention strategies within the country.

For peer review only

Methods

Study area and population

The study was conducted in school children living in three distinct ecological regions of Ecuador: the Andes highlands, the coastal lowlands, and the Amazon plains. The coastal region consists of six Provinces covering approximately 70,000 km^2 constituting less than a third of the surface area of Ecuador, but where 50% of the population lives.[9] The average annual temperature is between 24ºC and 26 ºC (18°C to 30°C) and the hottest period occurs during the rainy season from February through April. The climate is greatly influenced by the ocean currents, "El Nino" (warm) and Humboldt (cold). The land is generally low-lying with elevations below 800 meters above sea level (masl).[10 11] The Andean region of 11 Provinces bisects the country from North to South, covering about one fifth of land surface of Ecuador, and is inhabited by 44.5% of the population.[9] In this region, the altitude ranges from 1200 to 6000 masl and daily temperature varies between 3°C and 25°C depending on altitude. The dry season is between July and August and periods of high rainfall are generally seen between March and April and again in October.[10 11] The Amazon region consists of 6 Provinces and covers an area of 120,000 km^2 or about 50% of the land surface but is where only 5.1% of the population lives.[9] This region is characterized by an average of about 3000 mm of rainfall per year and an average annual temperature of 23ºC, high humidity, and relatively constant rainfall throughout the year.[10 11]

For peer review only

Study design and sample size

A cross-sectional study was conducted to estimate the prevalence and intensity of STH infections among rural school children at a national level. The study design followed WHO recommendations to allow comparison with other international studies.[12] The three ecologic regions constituted the strata for sampling. WHO recommendations are that 200-250 individuals in each zone or strata should be adequate to assess the need for control measures.[12] A list of all primary rural schools was compiled by the National Education Officer and six schools in each strata were selected at random. Private rural schools were included in the sampling although the vast majority was free-access government-funded schools. Fifty children attending the 5th grade (11-12 years of age) of primary education and who were present on the day of the survey were randomly selected in each school using a list of children provided by the school principal. If 50 were not present, we randomly selected children from other grades to complete the required number. Thus, overall we aimed to sample 300 children in each ecological zone to provide a total sample of 900 children.

Data collection

The field study was conducted between October 2011 and May 2012. Two data collection forms were adapted from WHO guidelines[12] to collect demographic, parasitological and anthropometric data from children and information about access to clean water, sanitation, distance to health care services and recent anthelmintic treatments provided in the schools.

For peer review only

Examination of stool samples

Single stool samples were collected from each child in plastic containers and examined for eggs and larvae using the standard Kato-Katz technique for soil-transmitted helminthes.[13] STH prevalence was expressed as the percentage of subjects found positive for each parasite and the prevalence of infection with at least one STH parasite. The number of eggs per gram of feces (EPG) was calculated by multiplying the egg count obtained by Kato-Katz by a conversion factor of 24. WHO guidelines were used to classify children as having light, moderate and heavy infections with each parasite.[14]

Anthropometric measurements

Height was measured without shoes using a portable stadiometer and weight with a digital scale (Filizola®, E-150/3P model). The instruments were calibrated periodically. All measurements were performed by trained personnel. Height-for-age z scores (HAZ) and Body Mass Index-for-age z scores (BAZ) were calculated using World Health Organization Child Growth Standards.[15] Children with height-for-age z scores and BMI-for-age z scores below to 2 standard deviation (<-2SD) were classified as stunted and thin/wasted, respectively. Children were classified as having malnutrition if they had at least one of these two conditions.

Statistical analysis

Data were double entered into Epi Info 7 and then exported into STATA v.10 for analysis. The prevalence, intensity of infection and 95% confidence intervals were estimated for each ecological zone. In a bivariate analysis, the chi-square test or Fisher's exact test ($p < 0.05$) was used to compare STH prevalence and infection intensity groups between

For peer review only

ecological zones. In multivariate analysis, the effect of geohelminth infections on malnutrition was assessed by logistic regression analysis with random effects to obtain robust standard errors taking into account the effect of clustering by school. OR and confidence intervals at 95% were estimated. Confounding factors controlled for in the analysis included sex, age, sewage, drinking water and recent anthelmintic treatment.

Results

Characteristics of study population

A total of 920 children from the three regions and 18 different schools were evaluated. All eligible children were evaluated and provided stool samples. Table 1 shows the characteristics of the study population. The mean age was 10.3 years (range 6-16) and there were slightly more girls than boys (51.2% vs. 48.8%). Children who participated in the study attended mostly Spanish-speaking schools (79.5 %). Access to piped water (52.4%) and a public sewer system (50.4%) in schools was present for approximately half the children evaluated. Most of children had received an anthelmintic drug during the previous 12 months (84.3%) and 70.5% had a health facility near their school (i.e. less than 1 km away).

For peer review only

Table 1. Characteristics of school-age children sampled in Ecuador, 2011-2012 (N=920)

Variables	N	%
Age (yrs.)		
7-10	450	48.9
11-16	470	51.1
Sex		
Male	449	48.8

Female	471	51.2
Rural Schools		
Public-Spanish speaking	731	79,5
Public/Municipal-Spanish	30	3,3
Municipal-Spanish	66	7,2
Public-Bilingual*	49	5,3
Private-Spanish	44	4,8
Drinking Water at schools		
Piped	482	52.4
Well	105	11,4
River or stream	333	36,2
Sewage at schools		
Public sewer system	464	50,4
Septic tank	379	41.2
Faeces to environment	77	8,4
Distance from school to a health service		
≤ 1 km	649	70.5
> 1 km	271	29.5
Last anthelmintic treatment in school		
≤ 6 months	208	22,6
7-12 months	568	61,7
> 12 months/none	144	15,7
Anthelmintic drug given in school		
Albendazol	625	67,9
Unknown	295	32,1
School location (Altitude)		
0-207 masl.	345	37.5
243-1869 masl.	315	34.2
≥2345 masl.	260	28.3

*Bilingual – Spanish and Quichua

For peer review only

Prevalence and Intensity of STH infection

The overall prevalence of infection with at least one of STH parasite was 27.9 % (95% CI, 25.0-30.8). Most frequent infections were with *T. trichiura* (19.3 %, 95% CI 16.8-21.9) followed by *A. lumbricoides* (18.5 %, 95% CI 16.0-21.0) and hookworm (5.0%, 95% CI 3.6-6.4). Forty-two percent of infected children had polyparasitism (i.e. 2 or 3 parasites)

(Table 2). The overall prevalence of high-intensity STH infections among infected children was 10.5% (95% CI 6.7-14-3), primarily for *A. lumbricoides* (14.7%, 95% CI 9.3-20.1) (Table 2).

Table 2. Prevalence and Intensity of Soil-Transmitted helminths in schoolchildren in Ecuador by region, 2011-2012.

	Coast n=303		Highlands n=308		Amazon n=309		Total N=920	
	n	% (95% CI)	n	% (95% CI)	n	% (95% CI)	n	% (95% CI)
**Prevalence of infection**								
Any geohelminth[a]	44	14.5 (0.1-18.5)	31	10.1 (6.7-13.4)	182	58.9 (53.0-64.0)	257	27.9 (25.0-30.8)
Ascaris lumbricoides[a]	13	4.3 (2.0-6.6)	23	7.5 (4.5-10.4)	134	43.4 (37.8-48.9)	170	18.5 (16.0-21.0)
Trichuris trichiura[a]	36	11.9 (8.2-15.5)	10	3.2 (1.3-5.2)	132	42.7 (37.2-48.3)	178	19.3 (16.8-21.9)
Hookworms[a]	0	0	1	0.3 (0-0.9)	45	14.6 (10.6-18.5)	46	5.0 (3.6-6.4)
**Mixed infections[a]**								
1 parasite	39	88.6 (78.9-98.4)	29	93.6 (84.4-100)	81	44.5 (37.2-51.8)	149	58.0 (51.9-64.1)
2 parasites	5	11.4 (1.6-21.1)	1	3.2 (0-9.8)	73	40.1 (32.9-47.3)	79	30.7 (25.1-36.4)
3 parasites	0	0	1	3.2 (0-9.8)	28	15.4 (10.1-20.7)	29	11.3 (7.4-15.2)
**Intensity of infection**								
Any helminth[a]								
Light	37	84.1 (73.1-95.1)	24	77.4 (62.4-92.5)	99	54.4 (47.1-61.7)	160	62.3 (56.3-68.2)
Moderate	6	13.6 (3.3-23.9)	7	22.6 (7.5-37.6)	57	31.3 (24.5-38.1)	70	27.2 (21.8-32.7)
Heavy	1	2.3 (0-6.7)	0	0	26	14.3 (9.2-19.4)	27	10.5 (6.7-14.3)
Ascaris lumbricoides[b]								
Light (1-4,999 epg)	9	69.2 (42.9-95.5)	16	69.6 (50.2-88.9)	54	40.3 (31.9-48.7)	79	46.5 (38.9-54.0)
Moderate (5,000-49,999 epg)	4	30.8 (4.5-57.1)	7	30.4 (11.1-49.8)	55	41.0 (32.6-49.5)	66	38.8 (31.4-46.2)
Heavy (>=50,000 epg)	0	0	0	0	25	18.7 (12.0-25.3)	25	14.7 (9.3-20.1)
Trichuris trichiura[c]								
Light	33	91.7	10	100	105	79.5	148	83.1

For peer review only

(1-999 epg)		(82.4-100.9)				(72.6-86.5)		(77.6-88.7)
Moderate	2	5.6	0	0	27	20.5	29	16.3
(1,000-9,999 epg)		(0-13.2)				(13.5-27.4)		(10.8-21.8)
Heavy	1	2.7	0	0	0	0	1	0.6
(>=10,000 epg)		(0-8.3)						(0-1.7)
Hookworms								
Light	0	0	1	100	41	91.1	42	91.3
(1-1,999 epg)						(82.5-99.7)		(82.8-99.8)
Moderate	0	0	0	0	3	6.7	3	6.5
(2,000-3,999 epg)						(0-14.2)		(0-13.9)
Heavy	0	0	0	0	1	2.2	1	2.2
(>=4,000 epg)						(0-6.7)		(0-6.5)

[a]*p* <0.001, [b]*p*<0.01, [c]*p*<0.05
epg – eggs per gram of feces

Figure 1 shows the age-prevalence and intensity patterns for *A. lumbricoides* and *T. trichiura*. The prevalence of *A. lumbricoides* infection varied little across age groups with the highest prevalence seen among 12 to 13 year olds. The prevalence of *T. trichiura* decreased slightly between 6 and 11 years and peaked at 14 to16 years, a variation that was statistically significant (p <0.001). The intensity of infection for both parasites followed similar patterns. Neither the prevalence nor intensity of infection differed significantly by sex for the two parasites (data not shown).

Prevalence and intensity of STH infections by region

Of the 18 selected schools, six were located in the Amazon region (n=309 children), six in the Highlands region (n=308 children) and six in the Coastal region (n=303 children). Significant differences in prevalence (p<0.0.01, for all STH infections) and intensity (p<0.01 for *A. lumbricoides* and p<0.05 for *T. trichiura*) were observed between the three regions. The highest rates of *A. lumbricoides*, *T. trichiura* and hookworm infection were in the Amazon region (43.4%, 42.7% and 14.6%, respectively). The other two regions showed

For peer review only

prevalence rates varying between 0 and 11.9% for each of the three parasites. Among infected children, heavy infection intensities with *A. lumbricoides* (18.7, 95% CI 12.0-25.3) and hookworm (2.2, 95% CI 0-6.7) were only detected in the Amazon region. In contrast, children with heavy infection intensities with *T. trichiura* were only found in the Coastal region (2.7, 95% CI 0-8.3) (Table 2 and Figure 2).

Prevalence of malnutrition

Table 3 summarizes the anthropometric findings of the children surveyed. The overall prevalence of stunting (HAZ <-2) and thinness/wasting (BAZ<-2) was 12.3% (95% CI, 10.2-14.4) and 2.1% (95% CI, 1.1-3.0), respectively. The percentage of children with malnutrition (at least one of the two conditions) was 14.2% (95% CI, 11.98-16.50). The prevalence of stunting varied significantly by region (p <0.001) with the highest prevalence observed in the Amazon region (18.8%, 95% CI 14.4-23.1). Children living in the Coastal region had the highest prevalence of thinness/wasting (3.3%, 95% CI 1.3-5.3); however, the differences between the three regions were not statistically significant (p=0.175) (Table 3).

Table 3. Anthropometric characteristics of survey of school children in Ecuador by region, 2011-2012

	**Total N=920**	**Coast n=303**	**Highlands n=308**	**Amazon n=309**	***p* value***
Mean BAZ (range)	0,31 (-3,43, 4.34)	0,32 (-3,43, 4,34)	0,29 (-3,41, 3,19)	0,31 (-2,73, 3,47)	0,960
Mean HAZ (range)	-0,79 (-6,95, 2,89)	-0,41 (-4,25, 2,88)	-0,73 (-3,58,2,89)	-1,22 (-6,95, 1,78)	<0.001
Wasting (%)§	19 (2,1)	10 (3,3)	4 (1,3)	5 (1,6)	0,175
Stunting (%)¶	113 (12,3)	18 (5,9)	37 (12,0)	58 (18,8)	<0.001
Malnutrition (%)**	131 (14,2)	27 (8,9)	41 (13,3)	63 (20,4)	<0.001

**p* value refers to significance of differences in prevalence between the 3 regions by ANOVA or X^2 testing.
HAZ- height-for-age z zcore; BAZ – BMI-for-age z score

§ Wasting: BAZ < -2

¶ Stunting: HAZ < -2
**Malnutrition: at least one of thinness/wasting or stunting

For peer review only

Association between STH infections and malnutrition

The results of bivariate and multivariate logistic regression analyses of the associations between STH infection and malnutrition are shown in Table 4. In bivariate analysis, the prevalence of both *A. lumbricoides* and *T. trichiura* were significantly associated with malnutrition (*A. lumbricoides:* 1.94, 95% CI 1.33-2.86; *T. trichiura*: 1.89, 95% CI 1.25-2.88), but were no longer significant after adjustment for potential confounders. Hookworm infection did not show a significant association with malnutrition in either bivariate or multivariate analyses.

There was some evidence of a dose-response relationship such that children with moderate and heavy-intensity infections with *A. lumbricoides* and *T. trichiura* infection more likely to have malnutrition than those children without infections (Table 4). However, only the association between heavy infection intensities with *A. lumbricoides* infection and malnutrition remained significant in multivariate analyses (adj. OR 3.7, 95% CI 1.48-9.24).

Table 4. Bivariate and Multivariate analysis of the associations between STH infection and malnutrition* among schoolchildren in Ecuador, 2011-2012.

STH infection	Total N=920	Malnutrition n (%)	OR (95% CI) crude	OR (95% CI)** adjusted	*p*
**Prevalence of infection**					
Any helminth					
Negative	663	78 (11.8)	1.0	1.0	
Positive	257	53 (20.6)	1.94 (1.33-2.86)	1.38 (0.88-2.14)	0.157
A. lumbricoides					
Negative	750	94 (12.5)	1.0	1.0	
Positive	170	37 (21.8)	1.94 (1.27-2.97)	1.37 (0.86-2.20)	0.190
T. trichiura					

For peer review only - http://bmjopen.bmj.com/site/about/guidelines.xhtml

Negative	742	93 (12.5)	1.0	1.0	
Positive	178	38 (21.4)	1.89 (1.25-2.88)	1.29 (0.79-2.10)	0.306
Hookworms					
Negative	874	120 (13.7)	1.0	1.0	
Positive	46	11 (23.9)	1.97 (0.98-3.99)	1.72 (0.84-3.51)	0.135
**Intensity of Infection**					
A. lumbricoides					
None	750	94 (12.5)	1.0	1.0	
Light	79	13 (16,5)	1.37 (0.73-2.59)	1.04 (0.54-2.02)	0.896
Moderate	66	14 (21,2)	1.88 (1.00-3.52)	1.25 (0.63-2.50)	0.526
Heavy	25	10 (40.0)	4.65 (2.03-10.66)	3.70 (1.48-9.24)	0.005
T. Trichiura					
None	742	93 (12.5)	1.0	1.0	
Light	148	29 (19.6)	1.70 (1.07-2.69)	1.19 (0.71-2.01)	0.506
Moderate/heavy	30	9 (30.0)	2.99 (1.33-6.73)	1.87 (0.76-4.60)	0.173
Hookworms					
None	874	120 (13.7)	1.0	1.0	
Light	42	9 (21,4%)	1.71 (0.80-3.67)	0.76 (0.32-1.79)	0.526
Moderate/heavy	4	2 (66,7%)	6.28 (0.88-45.03)	2.58 (0.33-20.18)	0.366

**OR (Odds Ratio) adjusted by sex, age, sewage, drinking water, anthelmintic treatment and clustering by school.
CI: Confidence Interval
*Any of thinness/wasting or stunting

Discussion

This study provides baseline data on the prevalence and intensity of STH infection and nutritional status in rural school children in the three ecologic regions of Ecuador. In this population, the overall prevalence of infection for at least one of the STH parasites was less than 50% and most infected children experienced light intensity infections. *T. trichuira* was the most prevalent parasite (19.3%) followed by *A. lumbricoides* (18.5%) and hookworm (5.0%). The present study found significant regional differences in the prevalence and intensity of STH infections the greatest prevalence of infection with each of the STH

For peer review only

investigated was observed in the Amazon region where about half of infected children had moderate to heavy infection intensities with *A. lumbricoides*. Malnutrition was observed in 14.1% of all children studied with the greatest prevalence seen in the sample from the Amazon region (20.4%).

The high prevalence of STH infection in the Amazon region (58.9 %) is comparable to the findings of other studies done in rural areas of the Ecuadorian Amazon that showed a prevalence of between 33.2% and 53.8%.[16-18] A study using a risk index for STH infection (based on census data such as overcrowding and lack of education and sanitation) to estimate STH prevalence within countries, showed high-risk and estimated prevalence (i.e. >50%) in the Amazon basin of Ecuador whereas prevalence outside these high risk pockets was estimated to be between 20 and 50%.[19] The concordance of observed and estimated prevalence in the Amazon region is unsurprising considering that the Ecuadorian Amazon is the poorest region in Ecuador. In 2014, poverty levels (based on unsatisfied basic needs) in the Amazon region reaches 45.9%, well above the national poverty rate (31.2%).[20] According to the Atlas of Socio-economic Inequalities of Ecuador,[21] the rural areas of the Amazon region are one of the areas in the country in which social conditions are considered to be critical. This area is characterized by deficiencies in housing and health indicators: 1/5 of households have safe water and 1/3 have adequate walls. Chronic malnutrition affects 27% of children aged 0-60 months.[22] These indicators confirm that STH are infections of poverty. Further, environmental and climatic conditions in the Amazon region are likely to be most favorable for the transmission of these parasites compared to the other two ecological region studied: the tropical, warm and humid climate throughout the year in the Amazon region is optimal for the development and survival of STH.[23 24]

In contrast, the infection rate of STH in the coastal region was relatively low compared to previous studies. Our results of a 14.5% prevalence for any STH differ from two studies conducted in the provinces of Manabí[25] and Esmeraldas[26 27] where STH prevalence was between 65% and 74.9%, respectively but with light or moderate infection intensities predominating. There are several possible explanations for these differences: i) the present study was designed to select a representative sample of schoolchildren in coastal Ecuador while the previous studies were directed at populations considered to be at high risk of STH infections; ii) the present survey was done in during 2011-2012 (after the introduction of systematic government-supported anthelmintic treatment programs, initiated in 2006 and targeted at school children) while the previous studies were done before the introduction of such programs and, therefore, might reflect the impact on prevalence of such school-based treatments; and iii) differences in diagnostic methods used or the number of stools collected from each child will affect diagnostic sensitivity – the Manabi study,[25] that also used the Kato-Katz to detect STH, collected three serial stool samples for STH detection which will have increased diagnostic sensitivity compared to a single sample as collected in our study. Diagnostic sensitivity in the Esmeraldas study would have been increased by the use of a concentration method in addition to Kato-Katz;[26] and iv) the overall prevalence and percentage of high intensity infections found in our study may have been underestimated because the majority of samples in this region were taken in the dry season when transmission is lower.

STH infections were of low prevalence (<20%) and light intensity in the Highland region where environmental conditions for the development of these STH are not optimal. Most of

For peer review only

the surveyed schools were located at altitudes between 1500 and 3000 metres where there is low humidity and temperatures range between 10 and 16 ° C limiting the transmission of these parasites. A low prevalence of STH was found also in a high altitude rural community (*A. lumbricoides:* 20%; *T. trichiura*: 6.4%) with mostly mild to moderate infection intensities.[28] Other studies made in subtropical areas of the highlands have shown a higher STH prevalence,[29] but were not areas selected for study in the present survey.

The nutritional status of school-age children in the study was characterized by significant levels of malnutrition (14.1%) with stunting being most frequent (12.3%), particularly among schoolchildren from the Amazon region. These data are in agreement with the results of the National Survey in Health and Nutrition which found levels of growth retardation of 15% in children between 5 to 11 years in Ecuador,[22] In this national survey, most districts in the Coastal and Amazon regions had a prevalence of stunting below 20% whereas most districts of the Highland region had stunting rates between 10% and 35% with the indigenous communities in the highlands being the most affected.[22] Our study observed a prevalence of stunting <20% in the highland region which is likely to be explained by the fact that our sample did not include predominantly indigenous populations.

Disease caused by STH is directly related to infection intensity.[30] We observed that children with severe *A. lumbricoides* infections were 3.7 more likely to have malnutrition than children without infection. This positive association was mainly for growth retardation measured by height-for-age z scores used as an indicator of chronic malnutrition and is in accordance with previous studies indicating stunting to be a common consequence of

For peer review only

STH.[31 32] There are several mechanisms by which STH could affect growth in children, including reduced food intake due to malabsorption and/or reduced appetite.[32] A study in northeast Brazil showed that in a cohort of children aged 2-7 years, helminthiasis acquired in early childhood was associated with a 4.6 cm shortfall in height by the age of 7.[33] In addition, a meta-analysis indicated that where the prevalence of intestinal nematodes is 50% or greater, anthelmintic drugs may have significant benefits including gains in weight, height, mid-upper arm circumference and skinfold thickness compared to untreated children[4].

Because we selected representative samples in each of the three ecologic zones, our findings are likely to be generalizable to schoolchildren of Ecuador and elsewhere in the Latin American region with similar levels of human development and geoclimatic characteristics. Based on our findings and in accordance with WHO guidelines,[12] we classify schoolchildren the Amazon region of Ecuador as being at high-risk for STH infections (prevalence of any STH $\geq$ 50%) and recommend that all school-age children (enrolled and non-enrolled) in this regions should be treated with anthelmintics twice a year. In Coastal and Highland regions (prevalence <20%), large-scale preventive chemotherapy is not recommended and affected individuals should be treated on a case-by-case basis. However, based on the findings of previous studies in these two regions, we recommend that subtropical and tropical areas within these regions where environmental conditions are propitious for STH transmission should also be considered at high risk and twice yearly anthelmintic treatments given to schoolchildren until more detailed local surveys can be done to better define STH infection risk within each Province. Long-term

For peer review only

interventions that should be implemented in parallel with chemotherapy include improved sanitation and water supply and health education.

A limitation of this study is the nature of a cross-sectional study that does not allow us to determine the temporal relationship between the presence of STH infection and malnutrition. The data from this study will help health authorities in Ecuador to develop an operational plan for the treatment of pre-school and school children in the three ecological regions in Ecuador. Clearly, efforts should be focused on the Amazon region where the prevalence is greater than 50% and where there are populations of marginalized and highly vulnerable groups such as indigenous groups. Similarly, these baseline data will allow health authorities to monitor the impact of control programs.

Conclusions

We have estimated the prevalence of STH infections in the three ecological zones in Ecuador and observed the highest prevalence and intensity of infection in the Amazon region, likely a consequence of poor living conditions and an environment that is highly favorable for transmission. The prevalence of stunting was higher also in the Amazon region and malnutrition was associated with the intensity of *A. lumbricoides* infections. There is a need for the implementation of deworming control programs for pre-school and school-age children with a focus on the Amazonian region. These programs should be integrated with other existing programs in the area with the aim of reducing parasite burdens to prevent potential adverse effects on the nutrition and health status of children.

Acknowledgments

For peer review only

We thank the Lcda. Silvia Erazo (Centro de salud del IESS-El Batán), Lcda. Jackeline Fonseca (Hospital Provincial Docente Ambato) and Lcda. Gabriela Andrade (Hospital San Vicente de Paul-Ibarra) for their help with laboratory analyses; members of the Ecuadorian Ministry of Public Health and the Pan-American Health Organization for technical support, and the Ecuadorian Ministry of Education for providing primary school databases. The school teachers, parents and children are thanked for their enthusiastic co-operation.

References

1. de Silva NR, Brooker S, Hotez PJ, Montresor A, Engels D, Savioli L. Soil-transmitted helminth infections: updating the global picture. *Trends Parasitol* 2003;19(12):547-51.
2. Pullan RL, Smith JL, Jasrasaria R, Brooker SJ. Global numbers of infection and disease burden of soil transmitted helminth infections in 2010. *Parasit Vectors* 2014;7:37.
3. WHO. Soil-transmitted helminthiases: estimates of the number of children needing preventive chemotherapy and number treated. *Wkly Epidemiol Rec* 2011;86:257-68.
4. Hall A, Hewitt G, Tuffrey V, de Silva N. A review and meta-analysis of the impact of intestinal worms on child growth and nutrition. *Matern Child Nutr* 2008;4 Suppl 1:118-236.
5. Dickson R, Awasthi S, Williamson P, Demellweek C, Garner P. Effects of treatment for intestinal helminth infection on growth and cognitive performance in children: systematic review of randomised trials. *BMJ* 2000;320(7251):1697-701.
6. WHO. Report of the 54th World Health Assembly. Control of Schistosomiasis and Soil-transmitted Helminth Infections. Geneva: WHO, 2001.
7. PAHO. Control y eliminacion de cinco enfermedades desatendidas en America Latina y el Caribem 2010-2015. Analisis de avances, prioridades y lineas de accion para filariasis linfatica, esquitosomiais, oncocercosis, tracoma y helmintiasis transmitidas por el contacto con el suelo. Washington, D.C.: PAHO, 2010.

For peer review only

8. Saboya MI, Catala L, Nicholls RS, Ault SK. Update on the mapping of prevalence and intensity of infection for soil-transmitted helminth infections in Latin America and the Caribbean: a call for action. *PLoS Negl Trop Dis* 2013;7(9):e2419.

9. Instituto Nacional de Estadísticas y Censo. Censo de Población y Vivienda en el Ecuador. 2010. http://www.inec.gob.ec/cpv/.

10. The Geography Site. http://www.geography-site.co.uk/pages/countries/climate/ecuador_climate.html/ (accessed 4 jul 2017).

11. Missouri Botanical Garden. http://www.mobot.org/MOBOT/research/ecuador/climates.shtml/ (accessed 25 jul 2017).

12. WHO. Helminth control in school-age children: a guide for managers of control programmes. 2nd. ed. Geneva: World Health Organization, 2011.

13. WHO. Diagnostic techniques for intesitnal parasitic infections (IPI) applicable to primary health care (PHC) services. Geneva: WHO, 1985.

14. WHO. Prevention and Control of Schistosomiasis and Soil-Transmitted helminthiasis. WHO Technical Series Report 912. Geneva: WHO, 2002.

15. de Onis M, Onyango AW, Borghi E, Siyam A, Nishida C, Siekmann J. Development of a WHO growth reference for school-aged children and adolescents. *Bull World Health Organ* 2007;85(9):660-7.

16. San Sebastian M, Santi S. Control of intestinal helminths in schoolchildren in Low-Napo, Ecuador: impact of a two-year chemotherapy program. *Rev Soc Bras Med Trop* 2000;33(1):69-73.

17. Cepon-Robins TJ, Liebert MA, Gildner TE, Urlacher SS, Colehour AM, Snodgrass JJ, et al. Soil-transmitted helminth prevalence and infection intensity among geographically and economically distinct Shuar communities in the Ecuadorian Amazon. *J Parasitol* 2014;100(5):598-607.

18. Romero-Sandoval N, Ortiz-Rico C, Sanchez-Perez HJ, Valdivieso D, Sandoval C, Pastor J, et al. Soil transmitted helminthiasis in indigenous groups. A community cross sectional study in the Amazonian southern border region of Ecuador. *BMJ Open* 2017;7(3):e013626.

For peer review only

19. Colston J, Saboya M. Soil-transmitted helminthiasis in Latin America and the Caribbean: modelling the determinants, prevalence, population at risk and costs of control at sub-national level. *Geospat Health* 2013;7(2):321-40.

20. Instituto Nacional de Estadisticas y Censo. Encuesta Nacional de Empleo, Desempleo y Subempleo-ENEMDU. 2014. http://www.inec.gob.ec.

21. SENPLADES. Atlas de las Desigualdades Socio-económicas del Ecuador. Quito: Secretaria Nacional de Planificación y Desarrollo, 2013.

22. Ministerio de Salud Pública del Ecuador. Encuesta Nacional de Salud y Nutrición ENSANUT-2012. Quito: Ministerio de Salud Publica, 2013.

23. O'Lorcain P, Holland CV. The public health importance of Ascaris lumbricoides. *Parasitology* 2000;121 Suppl:S51-71.

24. Stephenson LS, Holland CV, Cooper ES. The public health significance of Trichuris trichiura. *Parasitology* 2000;121 Suppl:S73-95.

25. Andrade C, Alava T, De Palacio IA, Del Poggio P, Jamoletti C, Gulletta M, et al. Prevalence and intensity of soil-transmitted helminthiasis in the city of Portoviejo (Ecuador). *Mem Inst Oswaldo Cruz* 2001;96(8):1075-9.

26. Moncayo AL, Vaca M, Amorim L, Rodriguez A, Erazo S, Oviedo G, et al. Impact of long-term treatment with ivermectin on the prevalence and intensity of soil-transmitted helminth infections. *PLoS Negl Trop Dis* 2008;2(9):e293.

27. Moncayo AL, Vaca M, Oviedo G, Erazo S, Quinzo I, Fiaccone RL, et al. Risk factors for atopic and non-atopic asthma in a rural area of Ecuador. *Thorax* 2010;65(5):409-16.

28. Rinne S, Rodas EJ, Galer-Unti R, Glickman N, Glickman LT. Prevalence and risk factors for protozoan and nematode infections among children in an Ecuadorian highland community. *Trans R Soc Trop Med Hyg* 2005;99(8):585-92.

29. Cooper PJ, Chico ME, Bland M, Griffin GE, Nutman TB. Allergic symptoms, atopy, and geohelminth infections in a rural area of Ecuador. *Am J Respir Crit Care Med* 2003;168(3):313-7.

30. Hotez PJ, Bundy DA, Beegle K, Brooker S, et.al. Helminth Infections: Soil-Transmitted Helminth Infections and Schistosomiasis. *Disease Control Priorities in Developing Countries*. 2nd. ed. Washington, D.C.: World Bank, 2006.

For peer review only

31. Stephenson LS, Latham MC, Ottesen EA. Malnutrition and parasitic helminth infections. *Parasitology* 2000;121 Suppl:S23-38.

32. Crompton DW, Nesheim MC. Nutritional impact of intestinal helminthiasis during the human life cycle. *Annu Rev Nutr* 2002;22:35-59.

33. Moore SR, Lima AA, Conaway MR, Schorling JB, Soares AM, Guerrant RL. Early childhood diarrhoea and helminthiases associate with long-term linear growth faltering. *Int J Epidemiol* 2001;30(6):1457-64.

Footnotes

Contributors: ALM, RL, and PJC conceived and designed the study. ALM collected data, wrote the statistical analysis plan, and cleaned and analyzed the data. PJC and ALM wrote the paper. All authors approved the final version of the manuscript.

Funding: Data collection was supported by Pan-American Health Organization. PJC was supported by Wellcome Trust grant 088862/Z/09/Z.

Competing interests: none declared

Patient consent: obtained

Ethics approval: The study was approved by the ethics committee of the Universidad Central del Ecuador, Quito, Ecuador. Informed written consent was obtained from a parent or guardian for each child. All children were offered treatment with a single dose of 400 mg of albendazole at the end of the study.

For peer review only

Provenance and peer review: Not commissioned; externally peer reviewed

Data sharing statement: No additional data are available

Figure Legends

Figure 1. Age-prevalence and age-intensity profiles (A and B, respectively) for *A. lumbricoides* (diamonds) and *T. trichiura* (squares) for schoolchildren. Shown are mean values and 95% confidence intervals.

Figure 2. Prevalence of soil-transmitted helminth infections in three ecological regions of Ecuador. Location of each school surveyed is marked. The size of the symbol corresponds to the % of children infected with STH in each school. The colors represent the three species found: Purple= *A. lumbricoides*, green= *T. trichiura* and red= hookworms.

For peer review only

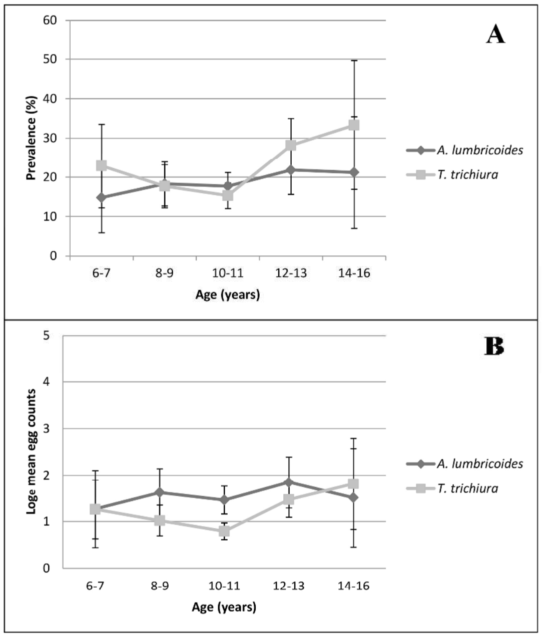

Figure 1

254x190mm (300 x 300 DPI)

For peer review only - http://bmjopen.bmj.com/site/about/guidelines.xhtml

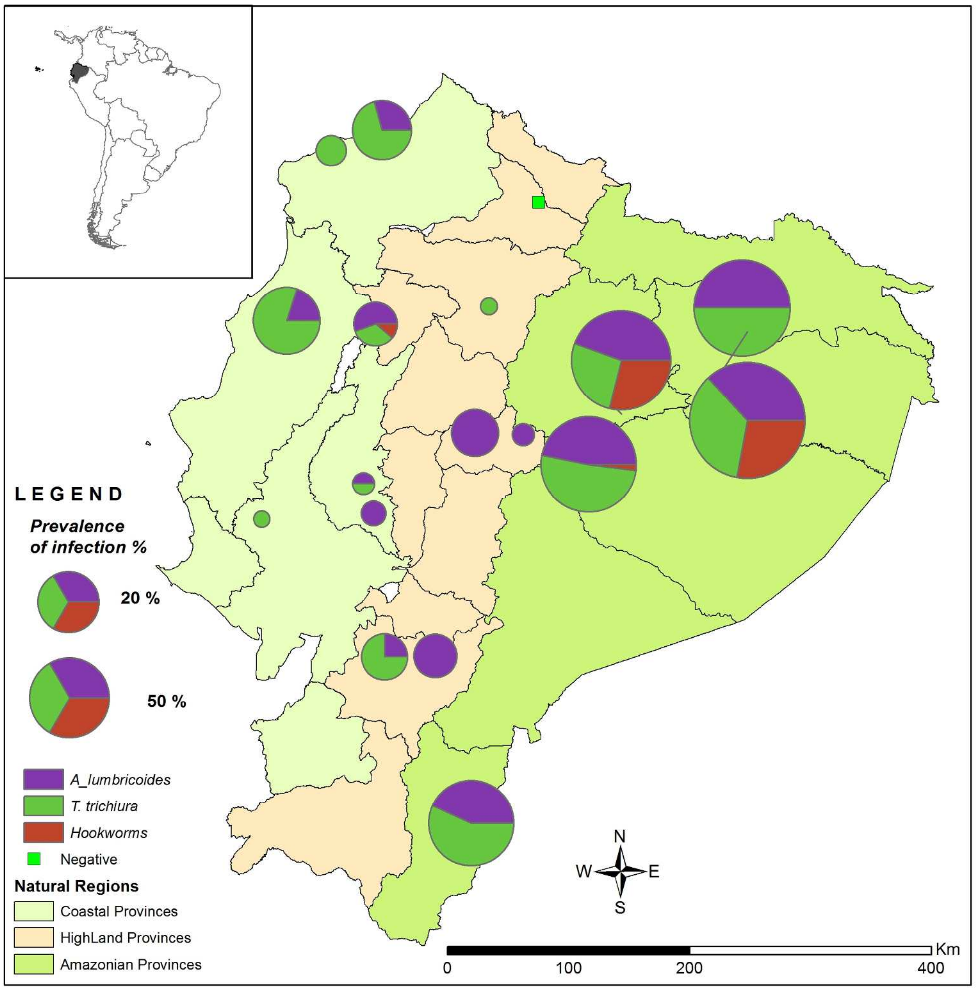

Figure 2

215x219mm (300 x 300 DPI)

STROBE Statement—Checklist of items that should be included in reports of ***cross-sectional studies***

	Item No	Recommendation	Page #
**Title and abstract**	1	(*a*) Indicate the study's design with a commonly used term in the title or the abstract	1
		(*b*) Provide in the abstract an informative and balanced summary of what was done and what was found	2,3
**Introduction**			
Background/rationale	2	Explain the scientific background and rationale for the investigation being reported	4,5
Objectives	3	State specific objectives, including any prespecified hypotheses	5
**Methods**			
Study design	4	Present key elements of study design early in the paper	7
Setting	5	Describe the setting, locations, and relevant dates, including periods of recruitment, exposure, follow-up, and data collection	6,7,8
Participants	6	(*a*) Give the eligibility criteria, and the sources and methods of selection of participants	7
Variables	7	Clearly define all outcomes, exposures, predictors, potential confounders, and effect modifiers. Give diagnostic criteria, if applicable	7,8,9
Data sources/ measurement	8*	For each variable of interest, give sources of data and details of methods of assessment (measurement). Describe comparability of assessment methods if there is more than one group	7,8
Bias	9	Describe any efforts to address potential sources of bias	7,8
Study size	10	Explain how the study size was arrived at	7
Quantitative variables	11	Explain how quantitative variables were handled in the analyses. If applicable, describe which groupings were chosen and why	8
Statistical methods	12	(*a*) Describe all statistical methods, including those used to control for confounding	8,9
		(*b*) Describe any methods used to examine subgroups and interactions	NA
		(*c*) Explain how missing data were addressed	
		(*d*) If applicable, describe analytical methods taking account of sampling strategy	8,9
		(*e*) Describe any sensitivity analyses	NA
**Results**			
Participants	13*	(a) Report numbers of individuals at each stage of study—eg numbers potentially eligible, examined for eligibility, confirmed eligible, included in the study, completing follow-up, and analysed	9
		(b) Give reasons for non-participation at each stage	9
		(c) Consider use of a flow diagram	NA
Descriptive data	14*	(a) Give characteristics of study participants (eg demographic, clinical, social) and information on exposures and potential confounders	9,10
		(b) Indicate number of participants with missing data for each variable of interest	9
Outcome data	15*	Report numbers of outcome events or summary measures	13
Main results	16	(*a*) Give unadjusted estimates and, if applicable, confounder-adjusted estimates and their precision (eg, 95% confidence interval). Make clear	14,15

For peer review only - http://bmjopen.bmj.com/site/about/guidelines.xhtml

		which confounders were adjusted for and why they were included	
		(*b*) Report category boundaries when continuous variables were categorized	11,12
		(*c*) If relevant, consider translating estimates of relative risk into absolute risk for a meaningful time period	NA
Other analyses	17	Report other analyses done—eg analyses of subgroups and interactions, and sensitivity analyses	NA
**Discussion**			
Key results	18	Summarise key results with reference to study objectives	15,16
Limitations	19	Discuss limitations of the study, taking into account sources of potential bias or imprecision. Discuss both direction and magnitude of any potential bias	19,20
Interpretation	20	Give a cautious overall interpretation of results considering objectives, limitations, multiplicity of analyses, results from similar studies, and other relevant evidence	16,17,18,19
Generalisability	21	Discuss the generalisability (external validity) of the study results	17
**Other information**			
Funding	22	Give the source of funding and the role of the funders for the present study and, if applicable, for the original study on which the present article is based	24

*Give information separately for exposed and unexposed groups.

Note: An Explanation and Elaboration article discusses each checklist item and gives methodological background and published examples of transparent reporting. The STROBE checklist is best used in conjunction with this article (freely available on the Web sites of PLoS Medicine at http://www.plosmedicine.org/, Annals of Internal Medicine at http://www.annals.org/, and Epidemiology at http://www.epidem.com/). Information on the STROBE Initiative is available at www.strobe-statement.org.

For peer review only

For peer review only - http://bmjopen.bmj.com/site/about/guidelines.xhtml

BMJ Open

Soil-transmitted helminth infections and nutritional status in Ecuador: findings from a national survey and implications for control strategies

Journal:	*BMJ Open*
Manuscript ID	bmjopen-2017-021319.R1
Article Type:	Research
Date Submitted by the Author:	08-Mar-2018
Complete List of Authors:	Moncayo, Ana-Lucia; Pontificia Universidad Catolica del Ecuador<br>Lovato, Raquel; Ministerio de Salud Publica<br>Cooper, Philip; St George's University of London, Institute of Infection and Immunity
**Primary Subject Heading**:	Infectious diseases
Secondary Subject Heading:	Global health, Public health, Epidemiology
Keywords:	soil-transmitted helminths, national survey, Ecuador, malnutrition

SCHOLARONE™
Manuscripts

Soil-transmitted helminth infections and nutritional status in Ecuador: findings from a national survey and implications for control strategies

Moncayo AL[1], Lovato R[2], Cooper PJ[3,4]

[1]Centro de Investigación Para la Salud en América Latina (CISeAL), Pontificia Universidad Católica del Ecuador, Quito, Ecuador
[2]Dirección de Vigilancia Epidemiológica, Ministerio de Salud Pública del Ecuador, Quito ,Ecuador
[3] Facultad de Ciencias Médicas, de la Salud y la Vida, Universidad Internacional del Ecuador, Quito, Ecuador.
[4]Institute of Infection and Immunity, St George's University of London, UK.

Correspondence to: amoncayo708@puce.edu.ec

For peer review only

Abstract

Objective The estimation of prevalence and intensity of soil-transmitted helminth infections (STH) at a country-level is an essential pre-requisite for the implementation of a rational control program. The aim of the present study was to estimate the prevalence and distribution of STH infections and malnutrition in school age children in rural areas of Ecuador.

Design Cross-sectional study from October 2011 and May 2012.

Setting Eighteen rural schools were randomly selected from the three ecological regions of Ecuador (coastal, highlands and Amazon basin).

Participants 920 children aged 6-16 years old.

Main outcome measures prevalence and intensity of STH infections associated with malnutrition (thinness/wasting or stunting)

Results: The results showed that 257 (27.9%) children were infected with at least one STH parasite. The prevalence of *Trichuris trichiura*, *Ascaris lumbricoides* and hookworm was 19.3%, 18.5%, and 5.0%, respectively. Malnutrition was present in 14.1 % of children and most common was stunting (12.3%). Compared to other regions, schoolchildren in the Amazon region had the highest STH prevalence (58.9%) of which a greater proportion of infections were moderate/heavy intensity (45.6%) and had the highest prevalence of malnutrition (14.1%). A positive association was observed between moderate to heavy infections with *A. lumbricoides* and malnutrition (adj. OR 1.85, 95% CI 1.04-3.31, p = 0.037).

For peer review only

Conclusions: Our estimate of the prevalence of STH infections of 27.9% at a national level in Ecuador is lower than suggested by previous studies. Our data indicate that schoolchildren living in the Amazon region have a greater risk of STH infection and stunting compared with children from other regions. The implementation of school-based preventive chemotherapy and nutritional supplement programmes within the Amazon region should be prioritized. Long-term control strategies require improvements in water, sanitation, and hygiene.

For peer review only

Strengths and limitations of this study

- This study is the first national survey of STH infections in schoolchildren in Ecuador and will inform the strategy for a national plan for deworming of schoolchildren in the three ecological regions in Ecuador.
- These baseline data will allow health authorities to monitor the impact of future deworming programmes among schoolchildren.
- The sampling strategy may have not detected localized geographic areas within the three regions with a higher prevalence of STH infections.
- The cross-sectional nature of the study does not allow us to determine the temporal relationship between the STH infections and malnutrition.
- Lack of a more complete nutritional evaluation including micronutrients.

For peer review only - http://bmjopen.bmj.com/site/about/guidelines.xhtml

Introduction

Soil-transmitted helminth infections (STH) (*Ascaris lumbricoides*, *Trichuris trichiura* and hookworm) are a major public health problem in tropical and sub-tropical regions of developing countries, especially in marginalized population with poor access to clean water and sanitation, and living in overcrowded conditions with low levels of education and lack of access to health services.[1] Recent estimates suggest that more than 1.45 billion humans worldwide are infected with STH parasites causing up to 4.98 million Years Lost due to Disability and 5.18 Disability-Adjusted Life Years.[2] Children are the group at highest risk of infection and most vulnerable to the pathologic consequences of infection. An estimated 13.9 million preschool and 35.4 million schoolchildren in Latin America and the Caribbean (LAC) were at risk of infection by STH in 2012.[3] STH infections are considered to have important deleterious effects on the nutritional status, growth, and physical development of infected children,[4 5] and may also affect cognitive performance and educability.[5]

In the last decade, an increasing number of international initiatives have established the aim to either reduce or to eliminate the disease burden caused by STHs and other helminth parasites prevalent in resource-poor regions. Chemotherapy still remains the most effective short-term approach for STH control in areas where infections are highly endemic. The World Health Assembly in 2001 urged all Member States where STH infections are endemic to treat at least 75% and up to 100% of all school-age children at risk of morbidity by 2010.[6] In Ecuador, international agencies and non-governmental organizations (NGOs) have formed partnerships with the government to provide deworming treatments for a large number of schoolchildren, and achieved the goal of at least 75% coverage of eligible

For peer review only - http://bmjopen.bmj.com/site/about/guidelines.xhtml

schoolchildren in 2006 and 2009, years for which data are available.[7] However, since 2009, there are no available data on treatment coverage of preschool and school-age children.

Few countries in LAC have implemented nationwide surveys on prevalence and intensity of STH infections in order to plan treatment strategies. A recent study identified gaps in available data on STH infections using data published between 2000 and 2010 in LAC.[8] A total of 335 published studies of STH prevalence were found in 18 countries: in Ecuador, 11 studies were analyzed of which 2 estimated a prevalence below 20%, 4 (36.4%) estimated a prevalence between 20-50% and 5 (45.5%) a prevalence of >50%. These data come from highly focal studies restricted to one or more parishes in 7 of the country's 24 provinces.

The aim of the present study was to carry out a national survey to estimate the current prevalence and intensity of STH infections and malnutrition in school age children. These data will be use to design and evaluate appropriate intervention strategies within the country.

Methods

Study area and population

The study was conducted in school children living in three distinct ecological regions of Ecuador: the Andes highlands, the coastal lowlands, and the Amazon plains. The coastal region consists of six Provinces covering approximately 70,000 km^2 constituting less than a third of the surface area of Ecuador, but where 50% of the population lives.[9] The average

For peer review only - http://bmjopen.bmj.com/site/about/guidelines.xhtml
For peer review only

annual temperature is between 24ºC and 26 ºC (18°C to 30°C) and the hottest period occurs during the rainy season from February through April. The climate is greatly influenced by the ocean currents, "El Nino" (warm) and Humboldt (cold). The land is generally low-lying with elevations below 800 meters above sea level (masl).[10 11] The Andean region of 11 Provinces bisects the country from North to South, covering about one fifth of land surface of Ecuador, and is inhabited by 44.5% of the population.[9] In this region, the altitude ranges from 1200 to 6000 masl and daily temperature varies between 3°C and 25°C depending on altitude. The dry season is between July and August and periods of high rainfall are generally seen between March and April and again in October.[10 11] The Amazon region consists of 6 Provinces and covers an area of 120,000 km^2 or about 50% of the land surface but is where only 5.1% of the population lives.[9] This region is characterized by an average of about 3000 mm of rainfall per year and an average annual temperature of 23ºC, high humidity, and relatively constant rainfall throughout the year.[10 11]

Poverty based on Unsatisfied Basic Needs was higher in the Amazon region (51.7%) than in the other regions (Coastal region: 40.1% and Andean region: 20.7%) in 2016[12]. Net enrollment on basic education and illiteracy rates were higher in Andean region (97.4%, 5.9%) than in Coastal (95.4%, 5.5%) and Amazon regions (95.7%, 5.0%).[12]

Study design and sample size

A cross-sectional study was conducted to estimate the prevalence and intensity of STH infections among rural school children at a national level. The study design followed WHO recommendations to allow comparison with other international studies.[13] The three ecologic regions constituted the strata for sampling. WHO recommendations are that 200-250

For peer review only - http://bmjopen.bmj.com/site/about/guidelines.xhtml

individuals in each zone or strata should be adequate to assess the need for control measures.[13] A list of all primary rural schools was compiled by the National Education Officer and six schools in each strata were selected at random. Private rural schools were included in the sampling although the vast majority was free-access government-funded schools. Fifty children attending the 5th grade (11-12 years of age) of primary education and who were present on the day of the survey were randomly selected in each school using a list of children provided by the school principal. If 50 were not present, we randomly selected children from other grades to complete the required number. Thus, overall we aimed to sample 300 children in each ecological zone to provide a total sample of 900 children.

Data collection

The field study was conducted between October 2011 and May 2012. Two data collection forms were adapted from WHO guidelines[13] to collect demographic, parasitological and anthropometric data from children and information about access to clean water and sanitation in schools, distance to health care services and recent anthelmintic treatments provided in the schools. Information on water supply, sanitation and anthelmintic treatment in schools was collected from teachers through an investigator-administered questionnaire and confirmed by direct observation.

Examination of stool samples

Single stool samples were collected from each child in plastic containers and examined for eggs using the standard Kato-Katz technique for soil-transmitted helminthes.[14] Kato-Katz slides were examined within 30-60 minutes of preparation. STH prevalence was expressed

For peer review only - http://bmjopen.bmj.com/site/about/guidelines.xhtml

as the percentage of children found positive for each parasite and the prevalence of infection with at least one STH parasite. The number of eggs per gram of feces (EPG) was calculated by multiplying the egg count obtained by Kato-Katz by a conversion factor of 24. WHO guidelines were used to classify children as having light, moderate and heavy infections with each parasite as follows:[15] for *A. lumbricoides*, light (1–4,999 epg), moderate (5,000-49,999 epg) and heavy (≥50,000 epg); for *T. trichiura,* light (1–999 epg), moderate (1,000-9,999 epg) and heavy (≥10,000 eggs/g stool); and for hookworm, light (1–1,999 eggs/g stool), moderate (2,000-3,999 epg) and heavy (>=4,000 epg).

Anthropometric measurements

Height was measured without shoes using a portable stadiometer and weight with a digital scale (Filizola®, E-150/3P model). The instruments were calibrated periodically. All measurements were performed by trained personnel. Height-for-age z scores (HAZ) and Body Mass Index-for-age z scores (BAZ) were calculated using World Health Organization Child Growth Standards.[16] Children with height-for-age z scores and BMI-for-age z scores below to 2 standard deviation (<-2SD) were classified as stunted and thin/wasted, respectively. Children were classified as having malnutrition if they had at least one of these two conditions.

Statistical analysis

Data were double entered into Epi Info 7 and then exported into STATA v.10 for analysis. The prevalence, intensity of infection and 95% confidence intervals were estimated for each ecological zone. The weighted prevalence for any helminth was also calculated using

For peer review only - http://bmjopen.bmj.com/site/about/guidelines.xhtml

the number of children aged 5-14 years in rural areas of each region. In a bivariate analysis, the chi-square test or Fisher's exact test ($p < 0.05$) was used to compare STH prevalence and infection intensity groups between ecological zones. In multivariate analysis, the association between geohelminth infections and malnutrition was assessed by logistic regression analysis with random effects to obtain robust standard errors taking into account the effect of clustering by school. OR and confidence intervals at 95% were estimated. Confounding factors controlled for in the analysis included sex, age, waste water disposal system, drinking water and recent anthelmintic treatment.

Ethics

The study protocol was approved by the ethics committee of the Universidad Central del Ecuador. Written informed consent was obtained from the parent of each child and verbal assent from each child to participate. All children were offered appropriate treatment.

Patient and Public Involvement

Patients were not involved in the design, recruitment and conduct of the study. The parent of each child received the results of the parasitological examination.

Results

Characteristics of study population

A total of 938 children from the three regions and 18 schools studied were eligible of which 920 (98.0%) were recruited and provided stool samples. Table 1 shows the characteristics of the study population. The mean age was 10.3 years (range 6-16) and there were slightly more girls than boys (51.2% vs. 48.8%). Children who participated in the study attended

For peer review only - http://bmjopen.bmj.com/site/about/guidelines.xhtml

mostly Spanish-speaking schools (79.5 %). Access to piped water (52.4%) and a public sewer system (50.4%) in schools was present for approximately half the children evaluated. Most of children had received an anthelmintic drug during the previous 12 months (84.3%) and 70.5% had a health facility near their school (i.e. less than 1 km away).

Table 1. Characteristics of school-age children sampled in Ecuador, 2011-2012 (N=920)

**Variables**	**N**	**%**
Age (yrs.)		
7-10	450	48.9
11-16	470	51.1
Sex		
Male	449	48.8
Female	471	51.2
Rural Schools		
Public-Spanish speaking	731	79,5
Public/Municipal-Spanish	30	3,3
Municipal-Spanish	66	7,2
Public-Bilingual*	49	5,3
Private-Spanish	44	4,8
Drinking Water at schools		
Piped	482	52.4
Well	105	11,4
River or stream	333	36,2
Sewage at schools		
Public sewer system	464	50,4
Septic tank	379	41.2
Faeces to environment	77	8,4
Distance from school to a health service		
≤ 1 km	649	70.5
> 1 km	271	29.5
Last anthelmintic treatment in school		
≤ 6 months	208	22,6
7-12 months	568	61,7
> 12 months/none	144	15,7
Anthelmintic drug given in school		
Albendazol	625	67,9
Unknown	295	32,1
School location (Altitude)		

For peer review only

For peer review only - http://bmjopen.bmj.com/site/about/guidelines.xhtml

0-207 masl.	345	37.5
243-1869 masl.	315	34.2
≥2345 masl.	260	28.3

*Bilingual – Spanish and Quichua

Prevalence and Intensity of STH infection

The overall prevalence of infection with at least one of STH parasite was 27.9 % (95% CI, 25.0-30.8) and the weighted prevalence was 17.6%. Most frequent infections were with *T. trichiura* (19.3 %, 95% CI 16.8-21.9) followed by *A. lumbricoides* (18.5 %, 95% CI 16.0-21.0) and hookworm (5.0%, 95% CI 3.6-6.4). Forty-two percent of infected children had polyparasitism (i.e. 2 or 3 parasites) (Table 2). The overall prevalence of moderate and high-intensity STH infections among infected children was 37.7% (95% CI 31.8-44-0), with the greatest proportions observed in the Amazon region (45.6%. 95% CI 38.2-53.1) and primarily consisting of infections with *A. lumbroides* (Table 2).

For peer review only

Table 2. Prevalence and Intensity of Soil-Transmitted helminths in schoolchildren in Ecuador by region, 2011-2012

	Coast n=303		Highlands n=308		Amazon n=309		Total N=920	
	n	% (95% CI)	n	% (95% CI)	n	% (95% CI)	n	% (95% CI)
**Prevalence of infection**								
Any geohelminth[a]	44	14.5 (0.1-18.5)	31	10.1 (6.7-13.4)	182	58.9 (53.0-64.0)	257	27.9 (25.0-30.8)
Ascaris lumbricoides[a]	13	4.3 (2.0-6.6)	23	7.5 (4.5-10.4)	134	43.4 (37.8-48.9)	170	18.5 (16.0-21.0)
Trichuris trichiura[a]	36	11.9 (8.2-15.5)	10	3.2 (1.3-5.2)	132	42.7 (37.2-48.3)	178	19.3 (16.8-21.9)
Hookworms[a]	0	0	1	0.3 (0-0.9)	45	14.6 (10.6-18.5)	46	5.0 (3.6-6.4)
**Mixed infections**[a]								
1 parasite	39	88.6 (78.9-98.4)	29	93.6 (84.4-100)	81	44.5 (37.2-51.8)	149	58.0 (51.9-64.1)
2 parasites	5	11.4 (1.6-21.1)	1	3.2 (0-9.8)	73	40.1 (32.9-47.3)	79	30.7 (25.1-36.4)
3 parasites	0	0	1	3.2 (0-9.8)	28	15.4 (10.1-20.7)	29	11.3 (7.4-15.2)
**Intensity of infection**								
Any helminth[a]								
Light	37	84.1 (73.1-95.1)	24	77.4 (62.4-92.5)	99	54.4 (47.1-61.7)	160	62.3 (56.3-68.2)
Moderate	6	13.6 (3.3-23.9)	7	22.6 (7.5-37.6)	57	31.3 (24.5-38.1)	70	27.2 (21.8-32.7)
Heavy	1	2.3 (0-6.7)	0	0	26	14.3 (9.2-19.4)	27	10.5 (6.7-14.3)
Moderate to heavy	7	15.9 (6.6-30.1)	7	22.6 (7.5-37.6)	83	45.6 (38.2-53.1)	97	37.7 (31.8-44.0)
Ascaris lumbricoides[b]								
Light	9	69.2 (42.9-95.5)	16	69.6 (50.2-88.9)	54	40.3 (31.9-48.7)	79	46.5 (38.9-54.0)

For peer review only - http://bmjopen.bmj.com/site/about/guidelines.xhtml
For peer review only

Moderate	4	30.8 (4.5-57.1)	7	30.4 (11.1-49.8)	55	41.0 (32.6-49.5)	66	38.8 (31.4-46.2)
Heavy	0	0	0	0	25	18.7 (12.0-25.3)	25	14.7 (9.3-20.1)
Trichuris trichiura[c]								
Light	33	91.7 (82.4-100.9)	10	100	105	79.5 (72.6-86.5)	148	83.1 (77.6-88.7)
Moderate	2	5.6 (0-13.2)	0	0	27	20.5 (13.5-27.4)	29	16.3 (10.8-21.8)
Heavy	1	2.7 (0-8.3)	0	0	0	0	1	0.6 (0-1.7)
Hookworms								
Light	0	0	1	100	41	91.1 (82.5-99.7)	42	91.3 (82.8-99.8)
Moderate	0	0	0	0	3	6.7 (0-14.2)	3	6.5 (0-13.9)
Heavy	0	0	0	0	1	2.2 (0-6.7)	1	2.2 (0-6.5)

[a] $p < 0.001$, [b] $p < 0.01$, [c] $p < 0.05$
epg – eggs per gram of feces

For peer review only

Age-prevalence and intensity patterns for *A. lumbricoides* and *T. trichiura* did not vary markedly by age in this sample of children aged 6 to 16 years although prevalence for both parasites was greater in older children (supplementary Figure S1). Neither prevalence nor intensity of infection differed significantly by sex for the two parasites (data not shown).

Prevalence and intensity of STH infections by region

Of the 18 selected schools, six were located in the Amazon region (n=309 children), six in the Highlands region (n=308 children) and six in the Coastal region (n=303 children). Significant differences in prevalence (p<0.0.01, for all STH infections) and intensity (p<0.01 for *A. lumbricoides* and p<0.05 for *T. trichiura*) were observed between the three regions. The highest rates of *A. lumbricoides*, *T. trichiura* and hookworm infection were in the Amazon region (43.4%, 42.7% and 14.6%, respectively). The other two regions showed prevalence rates varying between 0 and 11.9% for each of the three parasites. Among infected children, heavy infection intensities with *A. lumbricoides* (18.7, 95% CI 12.0-25.3) and hookworm (2.2, 95% CI 0-6.7) were only detected in the Amazon region. In contrast, children with heavy infection intensities with *T. trichiura* were only found in the Coastal region (2.7, 95% CI 0-8.3) (Table 2 and Figure 1).

Prevalence of malnutrition

Table 3 summarizes the anthropometric findings of the children surveyed. The overall prevalence of stunting (HAZ <-2) and thinness/wasting (BAZ<-2) was 12.3% (95% CI, 10.2-14.4) and 2.1% (95% CI, 1.1-3.0), respectively. The percentage of children with malnutrition (at least one of the two conditions) was 14.2% (95% CI, 11.98-16.50). The

For peer review only - http://bmjopen.bmj.com/site/about/guidelines.xhtml

prevalence of stunting varied significantly by region (p <0.001) with the highest prevalence observed in the Amazon region (18.8%, 95% CI 14.4-23.1). Children living in the Coastal region had the highest prevalence of thinness/wasting (3.3%, 95% CI 1.3-5.3); however, the differences between the three regions were not statistically significant (p=0.175) (Table 3).

Table 3. Nutritional and anthropometric characteristics of sampled Ecuadorian school children by region, 2011-2012.

	Total N=920	Coast n=303	Highlands n=308	Amazon n=309	*p* value*
Mean BAZ (range)	0,31 (-3,43, 4.34)	0,32 (-3,43, 4,34)	0,29 (-3,41, 3,19)	0,31 (-2,73, 3,47)	0,960
Mean HAZ (range)	-0,79 (-6,95, 2,89)	-0,41 (-4,25, 2,88)	-0,73 (-3,58,2,89)	-1,22 (-6,95, 1,78)	<0.001
Wasting (%)§	19 (2,1)	10 (3,3)	4 (1,3)	5 (1,6)	0,175
Stunting (%)¶	113 (12,3)	18 (5,9)	37 (12,0)	58 (18,8)	<0.001
Malnutrition (%)**	131 (14,2)	27 (8,9)	41 (13,3)	63 (20,4)	<0.001

**p* value refers to significance of differences in prevalence between the 3 regions by ANOVA or X^2 testing.
HAZ- height-for-age z zcore; BAZ – BMI-for-age z score
§ Wasting: BAZ < -2
¶ Stunting: HAZ < -2
**Malnutrition: at least one of thinness/wasting or stunting

Association between STH infections and malnutrition

The results of bivariate and multivariate logistic regression analyses of the associations between STH infection and malnutrition are shown in Table 4. In bivariate analysis, the prevalence of both *A. lumbricoides* and *T. trichiura* were significantly associated with malnutrition (*A. lumbricoides:* 1.94, 95% CI 1.33-2.86; *T. trichiura*: 1.89, 95% CI 1.25-2.88), but were no longer significant after adjustment for potential confounders. Hookworm infection did not show a significant association with malnutrition in either bivariate or multivariate analyses.

For peer review only - http://bmjopen.bmj.com/site/about/guidelines.xhtml

There was some evidence of a dose-response relationship such that children with moderate to heavy-intensity infections with *A. lumbricoides* and *T. trichiura* were more likely to have malnutrition than those children without infections (Table 4). However, only the association between moderate to heavy infection intensities with *A. lumbricoides* and malnutrition remained significant in multivariate analyses (adj. OR 1.85, 95% CI 1.04-3.31).

For peer review only

Table 4. Bivariate and Multivariate analysis of the associations between STH infection and malnutrition* among schoolchildren in Ecuador, 2011-2012.

STH infection	Total N=920	Malnutrition n (%)	OR (95% CI) crude	OR (95% CI)** adjusted	*p*
**Prevalence of infection**					
Any helminth					
Negative	663	78 (11.8)	1.0	1.0	
Positive	257	53 (20.6)	1.94 (1.33-2.86)	1.38 (0.88-2.14)	0.157
A. lumbricoides					
Negative	750	94 (12.5)	1.0	1.0	
Positive	170	37 (21.8)	1.94 (1.27-2.97)	1.37 (0.86-2.20)	0.190
T. trichiura					
Negative	742	93 (12.5)	1.0	1.0	
Positive	178	38 (21.4)	1.89 (1.25-2.88)	1.29 (0.79-2.10)	0.306
Hookworms					
Negative	874	120 (13.7)	1.0	1.0	
Positive	46	11 (23.9)	1.97 (0.98-3.99)	1.72 (0.84-3.51)	0.135
**Intensity of Infection**					
A. lumbricoides					
None	750	94 (12.5)	1.0	1.0	
Light	79	13 (16,5)	1.37 (0.73-2.59)	1.04 (0.54-2.02)	0.896
Moderate/heavy	91	24 (26.4)	2.50 (1.49-4.18)	1.85 (1.04-3.31)	0.037
T. Trichiura					
None	742	93 (12.5)	1.0	1.0	
Light	148	29 (19.6)	1.70 (1.07-2.69)	1.19 (0.71-2.01)	0.506
Moderate/heavy	30	9 (30.0)	2.99 (1.33-6.73)	1.87 (0.76-4.60)	0.173

For peer review only - http://bmjopen.bmj.com/site/about/guidelines.xhtml

Hookworms					
None	874	120 (13.7)	1.0	1.0	
Light	42	9 (21,4%)	1.71 (0.80-3.67)	0.76 (0.32-1.79)	0.526
Moderate/heavy	4	2 (66,7%)	6.28 (0.88-45.03)	2.58 (0.33-20.18)	0.366

**OR (Odds Ratio) adjusted by sex, age, sewage, drinking water, anthelmintic treatment and clustering by school.
CI: Confidence Interval
*Any of thinness/wasting or stunting

Discussion

This study provides baseline data on the prevalence and intensity of STH infection and nutritional status in rural school children in the three ecologic regions of Ecuador. In this population, the overall prevalence of infection for at least one of the STH parasites was less than 50% and most infected children experienced light intensity infections. *T. trichuira* was the most prevalent parasite (19.3%) followed by *A. lumbricoides* (18.5%) and hookworm (5.0%). The present study found significant regional differences in the prevalence and intensity of STH infections the greatest prevalence of infection with each of the STH investigated was observed in the Amazon region where about half of infected children had moderate to heavy infection intensities with *A. lumbricoides*. Malnutrition was observed in 14.1% of all children studied with the greatest prevalence seen in the sample from the Amazon region (20.4%).

A systematic review of studies of prevalence of soil-transmitted helminth infection in South America from 2005-2012 estimated a prevalence of 28.1% for Ecuador, similar to the prevalence found in our study. Prevalence rates below 20% were reported for Argentina (18.9%) and Uruguay (18.8%) whereas the highest prevalence rates were reported for

For peer review only - http://bmjopen.bmj.com/site/about/guidelines.xhtml

For peer review only

French Guyana (46.2%) and Surinam (40.1%). The remaining countries showed prevalence rates of between 25% and 39%.[17]

The high prevalence of STH infection in the Amazon region (58.9 %) is comparable to the findings of other studies done in rural areas of the Ecuadorian Amazon that showed a prevalence of between 33.2% and 53.8%.[18-20] A study using a risk index for STH infection (based on census data such as overcrowding and lack of education and sanitation) to estimate STH prevalence within countries, showed high-risk and estimated prevalence (i.e. >50%) in the Amazon basin of Ecuador whereas prevalence outside these high risk pockets was estimated to be between 20 and 50%.[21] The concordance of observed and estimated prevalence in the Amazon region is unsurprising considering that the Ecuadorian Amazon is the poorest region in Ecuador. In 2016 , poverty levels (based on unsatisfied basic needs) in the Amazon region were 51.7%, well above the national poverty rate of 32.0%.[12] According to the Atlas of Socio-economic Inequalities of Ecuador,[22] the rural areas of the Amazon region are one of the areas in the country in which social conditions are considered to be critical. This area is characterized by deficiencies in housing and health indicators: 1/5 of households have safe water and 1/3 have adequate walls. Chronic malnutrition affects 27% of children aged 0-60 months.[23] These indicators confirm that STH are infections of poverty. Further, environmental and climatic conditions in the Amazon region are likely to be most favorable for the transmission of these parasites compared to the other two ecological region studied: the tropical, warm and humid climate throughout the year in the Amazon region is optimal for the development and survival of STH.[24 25]

For peer review only - http://bmjopen.bmj.com/site/about/guidelines.xhtml

In contrast, the infection rate of STH in the coastal region was relatively low compared to previous studies. Our results of a 14.5% prevalence for any STH differ from two studies conducted in the provinces of Manabí[26] and Esmeraldas[27 28] where STH prevalence was between 65% and 74.9%, respectively but with light or moderate infection intensities predominating. There are several possible explanations for these differences: i) the present study was designed to select a representative sample of schoolchildren in coastal Ecuador while the previous studies were directed at populations considered to be at high risk of STH infections; ii) the present survey was done in during 2011-2012 (after the introduction of systematic government-supported anthelmintic treatment programs, initiated in 2006 and targeted at school children) while the previous studies were done before the introduction of such programs and, therefore, might reflect the impact on prevalence of such school-based treatments; and iii) differences in diagnostic methods used or the number of stools collected from each child will affect diagnostic sensitivity – the Manabi study,[26] that also used the Kato-Katz to detect STH, collected three serial stool samples for STH detection which will have increased diagnostic sensitivity compared to a single sample as collected in our study. Diagnostic sensitivity in the Esmeraldas study would have been increased by the use of a concentration method in addition to Kato-Katz;[27] and iv) the overall prevalence and percentage of high intensity infections found in our study may have been underestimated because the majority of samples in this region were taken in the dry season when transmission is lower.

STH infections were of low prevalence (<20%) and light intensity in the Highland region where environmental conditions for the development of these STH are not optimal. Most of the surveyed schools were located at altitudes between 1500 and 3000 metres where there is

For peer review only - http://bmjopen.bmj.com/site/about/guidelines.xhtml

low humidity and temperatures range between 10 and 16 ° C limiting the transmission of these parasites. A low prevalence of STH was found also in a high altitude rural community (*A. lumbricoides:* 20%; *T. trichiura*: 6.4%) with mostly mild to moderate infection intensities.[29] Other studies made in subtropical areas of the highlands have shown a higher STH prevalence,[30] but were not areas selected for study in the present survey.

The nutritional status of school-age children in the study was characterized by significant levels of malnutrition (14.1%) with stunting being most frequent (12.3%), particularly among schoolchildren from the Amazon region. These data are in agreement with the results of the National Survey in Health and Nutrition which found levels of growth retardation of 15% in children between 5 to 11 years in Ecuador,[23] In this national survey, most districts in the Coastal and Amazon regions had a prevalence of stunting below 20% whereas most districts of the Highland region had stunting rates between 10% and 35% with the indigenous communities in the highlands being the most affected.[23] Our study observed a prevalence of stunting <20% in the highland region which is likely to be explained by the fact that our sample did not include predominantly indigenous populations.

Disease caused by STH is directly related to infection intensity.[31] We observed that children with severe *A. lumbricoides* infections were 3.7 more likely to have malnutrition than children without infection. This positive association was mainly for growth retardation measured by height-for-age z scores used as an indicator of chronic malnutrition and is in accordance with previous studies indicating stunting to be a common consequence of STH.[32 33] There are several mechanisms by which STH could affect growth in children,

For peer review only - http://bmjopen.bmj.com/site/about/guidelines.xhtml

For peer review only

including reduced food intake due to malabsorption and/or reduced appetite.[33] A study in northeast Brazil showed that in a cohort of children aged 2-7 years, helminthiasis acquired in early childhood was associated with a 4.6 cm shortfall in height by the age of 7.[34] In addition, a meta-analysis indicated that where the prevalence of intestinal nematodes is 50% or greater, anthelmintic drugs may have significant benefits including gains in weight, height, mid-upper arm circumference and skinfold thickness compared to untreated children[4].

Because we selected representative samples in each of the three ecologic zones, our findings are likely to be generalizable to schoolchildren of Ecuador and elsewhere in the Latin American region with similar levels of human development and geoclimatic characteristics. Based on our findings and in accordance with WHO guidelines,[13] we classify schoolchildren the Amazon region of Ecuador as being at high-risk for STH infections (prevalence of any STH ≥ 50%) and recommend that all school-age children (enrolled and non-enrolled) in this regions should be treated with anthelmintics twice a year. In Coastal and Highland regions (prevalence <20%, current indications recommend no large-scale preventive chemotherapy and treatment of affected individuals on a case-by-case basis. However, given that this survey was done in the context of previous interventions with anthelmintics it would be prudent to continue annual treatments to prevent a potential rebound in prevalence until repeat surveys are able to confirm limited transmission in these regions. In addition, because previous studies have shown high prevalence areas in these two regions, we recommend that subtropical and tropical areas within these regions where environmental conditions are propitious for STH transmission should also be considered at high risk and twice yearly anthelmintic treatments given to

For peer review only - http://bmjopen.bmj.com/site/about/guidelines.xhtml
For peer review only

schoolchildren until more detailed local surveys can be done to better define STH infection risk within each province. Long-term interventions that should be implemented in parallel with chemotherapy include improved sanitation and water supply and health education.

A limitation of this study is the nature of a cross-sectional study that does not allow us to determine the temporal relationship between the presence of STH infection and malnutrition. In addition, a single stool sample may underestimate STH prevalence, especially for hookworm infection. The data from this study will help health authorities in Ecuador to develop an operational plan for the treatment of pre-school and school children in the three ecological regions in Ecuador. Clearly, efforts should be focused on the Amazon region where the prevalence is greater than 50% and where there are populations of marginalized and highly vulnerable groups such as indigenous groups. Similarly, these baseline data will allow health authorities to monitor the impact of control programs. Although this study was carried out in 2011-2012, a national deworming programme has not yet been launched in Ecuador.

Conclusions

We have estimated the prevalence of STH infections in the three ecological zones in Ecuador and observed the highest prevalence and intensity of infection in the Amazon region, likely a consequence of poor living conditions and an environment that is highly favorable for transmission. The prevalence of stunting was higher also in the Amazon region and malnutrition was associated with the intensity of *A. lumbricoides* infections. There is a need for the implementation of deworming control programs combined with

For peer review only - http://bmjopen.bmj.com/site/about/guidelines.xhtml

interventions to improve nutrition for pre-school and school-age children with a focus on the Amazon region. These programs should be integrated with other existing programs with the aim of reducing parasite burdens to prevent potential adverse effects on the nutritional and health status of children. Long-term strategies require improvements in drinking water, sanitation, and hygiene

Acknowledgments

We thank the Lcda. Silvia Erazo (Centro de salud del IESS-El Batán), Lcda. Jackeline Fonseca (Hospital Provincial Docente Ambato) and Lcda. Gabriela Andrade (Hospital San Vicente de Paul-Ibarra) for their help with laboratory analyses; members of the Ecuadorian Ministry of Public Health and the Pan-American Health Organization for technical support; the Ecuadorian Ministry of Education for providing primary school databases, and César Yumiseva for your help with the map. The school teachers, parents and children are thanked for their enthusiastic co-operation.

References

1. de Silva NR, Brooker S, Hotez PJ, Montresor A, Engels D, Savioli L. Soil-transmitted helminth infections: updating the global picture. *Trends Parasitol* 2003;19(12):547-51.

2. Pullan RL, Smith JL, Jasrasaria R, Brooker SJ. Global numbers of infection and disease burden of soil transmitted helminth infections in 2010. *Parasit Vectors* 2014;7:37.

3. WHO. Soil-transmitted helminthiases: estimates of the number of children needing preventive chemotherapy and number treated. *Wkly Epidemiol Rec* 2011;86:257-68.

For peer review only - http://bmjopen.bmj.com/site/about/guidelines.xhtml

For peer review only

4. Hall A, Hewitt G, Tuffrey V, de Silva N. A review and meta-analysis of the impact of intestinal worms on child growth and nutrition. *Matern Child Nutr* 2008;4 Suppl 1:118-236.

5. Dickson R, Awasthi S, Williamson P, Demellweek C, Garner P. Effects of treatment for intestinal helminth infection on growth and cognitive performance in children: systematic review of randomised trials. *BMJ* 2000;320(7251):1697-701.

6. WHO. Report of the 54th World Health Assembly. Control of Schistosomiasis and Soil-transmitted Helminth Infections. Geneva: WHO, 2001.

7. PAHO. Control y eliminacion de cinco enfermedades desatendidas en America Latina y el Caribem 2010-2015. Analisis de avances, prioridades y lineas de accion para filariasis linfatica, esquitosomiais, oncocercosis, tracoma y helmintiasis transmitidas por el contacto con el suelo. Washington, D.C.: PAHO, 2010.

8. Saboya MI, Catala L, Nicholls RS, Ault SK. Update on the mapping of prevalence and intensity of infection for soil-transmitted helminth infections in Latin America and the Caribbean: a call for action. *PLoS Negl Trop Dis* 2013;7(9):e2419.

9. Instituto Nacional de Estadísticas y Censo. Censo de Población y Vivienda en el Ecuador. 2010. http://www.inec.gob.ec/cpv/.

10. The Geography Site. http://www.geography-site.co.uk/pages/countries/climate/ecuador_climate.html/ (accessed 4 jul 2017).

11. Missouri Botanical Garden. http://www.mobot.org/MOBOT/research/ecuador/climates.shtml/ (accessed 25 jul 2017).

12. Instituto Nacional de Estadisticas y Censo. Encuesta Nacional de Empleo, Desempleo y Subempleo-ENEMDU. 2016. http://www.inec.gob.ec.

For peer review only

For peer review only - http://bmjopen.bmj.com/site/about/guidelines.xhtml

13. WHO. Helminth control in school-age children: a guide for managers of control programmes. 2nd. ed. Geneva: World Health Organization, 2011.

14. WHO. Diagnostic techniques for intesitnal parasitic infections (IPI) applicable to primary health care (PHC) services. Geneva: WHO, 1985.

15. WHO. Prevention and Control of Schistosomiasis and Soil-Transmitted helminthiasis. WHO Technical Series Report 912. Geneva: WHO, 2002.

16. de Onis M, Onyango AW, Borghi E, Siyam A, Nishida C, Siekmann J. Development of a WHO growth reference for school-aged children and adolescents. *Bull World Health Organ* 2007;85(9):660-7.

17. Chammartin F, Scholte RG, Guimaraes LH, Tanner M, Utzinger J, Vounatsou P. Soil-transmitted helminth infection in South America: a systematic review and geostatistical meta-analysis. *Lancet Infect Dis* 2013;13(6):507-18.

18. San Sebastian M, Santi S. Control of intestinal helminths in schoolchildren in Low-Napo, Ecuador: impact of a two-year chemotherapy program. *Rev Soc Bras Med Trop* 2000;33(1):69-73.

19. Cepon-Robins TJ, Liebert MA, Gildner TE, Urlacher SS, Colehour AM, Snodgrass JJ, et al. Soil-transmitted helminth prevalence and infection intensity among geographically and economically distinct Shuar communities in the Ecuadorian Amazon. *J Parasitol* 2014;100(5):598-607.

20. Romero-Sandoval N, Ortiz-Rico C, Sanchez-Perez HJ, Valdivieso D, Sandoval C, Pastor J, et al. Soil transmitted helminthiasis in indigenous groups. A community cross sectional study in the Amazonian southern border region of Ecuador. *BMJ Open* 2017;7(3):e013626.

For peer review only - http://bmjopen.bmj.com/site/about/guidelines.xhtml

21. Colston J, Saboya M. Soil-transmitted helminthiasis in Latin America and the Caribbean: modelling the determinants, prevalence, population at risk and costs of control at sub-national level. *Geospat Health* 2013;7(2):321-40.

22. SENPLADES. Atlas de las Desigualdades Socio-económicas del Ecuador. Quito: Secretaria Nacional de Planificación y Desarrollo, 2013.

23. Ministerio de Salud Pública del Ecuador. Encuesta Nacional de Salud y Nutrición ENSANUT-2012. Quito: Ministerio de Salud Publica, 2013.

24. O'Lorcain P, Holland CV. The public health importance of Ascaris lumbricoides. *Parasitology* 2000;121 Suppl:S51-71.

25. Stephenson LS, Holland CV, Cooper ES. The public health significance of Trichuris trichiura. *Parasitology* 2000;121 Suppl:S73-95.

26. Andrade C, Alava T, De Palacio IA, Del Poggio P, Jamoletti C, Gulletta M, et al. Prevalence and intensity of soil-transmitted helminthiasis in the city of Portoviejo (Ecuador). *Mem Inst Oswaldo Cruz* 2001;96(8):1075-9.

27. Moncayo AL, Vaca M, Amorim L, Rodriguez A, Erazo S, Oviedo G, et al. Impact of long-term treatment with ivermectin on the prevalence and intensity of soil-transmitted helminth infections. *PLoS Negl Trop Dis* 2008;2(9):e293.

28. Moncayo AL, Vaca M, Oviedo G, Erazo S, Quinzo I, Fiaccone RL, et al. Risk factors for atopic and non-atopic asthma in a rural area of Ecuador. *Thorax* 2010;65(5):409-16.

29. Rinne S, Rodas EJ, Galer-Unti R, Glickman N, Glickman LT. Prevalence and risk factors for protozoan and nematode infections among children in an Ecuadorian highland community. *Trans R Soc Trop Med Hyg* 2005;99(8):585-92.

For peer review only

For peer review only - http://bmjopen.bmj.com/site/about/guidelines.xhtml

30. Cooper PJ, Chico ME, Bland M, Griffin GE, Nutman TB. Allergic symptoms, atopy, and geohelminth infections in a rural area of Ecuador. *Am J Respir Crit Care Med* 2003;168(3):313-7.

31. Hotez PJ, Bundy DA, Beegle K, Brooker S, et.al. Helminth Infections: Soil-Transmitted Helminth Infections and Schistosomiasis. *Disease Control Priorities in Developing Countries*. 2nd. ed. Washington, D.C.: World Bank, 2006.

32. Stephenson LS, Latham MC, Ottesen EA. Malnutrition and parasitic helminth infections. *Parasitology* 2000;121 Suppl:S23-38.

33. Crompton DW, Nesheim MC. Nutritional impact of intestinal helminthiasis during the human life cycle. *Annu Rev Nutr* 2002;22:35-59.

34. Moore SR, Lima AA, Conaway MR, Schorling JB, Soares AM, Guerrant RL. Early childhood diarrhoea and helminthiases associate with long-term linear growth faltering. *Int J Epidemiol* 2001;30(6):1457-64.

For peer review only

Footnotes

Contributors: ALM, RL, and PJC conceived and designed the study. ALM collected data, wrote the statistical analysis plan, and cleaned and analyzed the data. PJC and ALM wrote the paper. All authors approved the final version of the manuscript.

Funding: Data collection was supported by Pan-American Health Organization. PJC was supported by Wellcome Trust grant 088862/Z/09/Z.

For peer review only - http://bmjopen.bmj.com/site/about/guidelines.xhtml

Competing interests: none declared

Patient consent: obtained

Ethics approval: The study was approved by the ethics committee of the Universidad Central del Ecuador, Quito, Ecuador. Informed written consent was obtained from a parent or guardian for each child. All children were offered treatment with a single dose of 400 mg of albendazole at the end of the study.

Provenance and peer review: Not commissioned; externally peer reviewed

Data sharing statement: All data relating to the study are summarised in the article. Access to the original data can be obtained through the corresponding author.

Figure Legends

Figure 1. Prevalence of soil-transmitted helminth infections in three ecological regions of Ecuador. Location of each school surveyed is marked. The size of the symbol corresponds to the % of children infected with STH in each school. The colors represent the three species found: Purple= *A. lumbricoides*, green= *T. trichiura* and red= hookworms.

Supplementary Figure legends

For peer review only - http://bmjopen.bmj.com/site/about/guidelines.xhtml

For peer review only

Figure S1. Age-prevalence and age-intensity profiles (A and B, respectively) for *A. lumbricoides* (diamonds) and *T. trichiura* (squares) for schoolchildren. Shown are mean values and 95% confidence intervals.

For peer review only

For peer review only - http://bmjopen.bmj.com/site/about/guidelines.xhtml

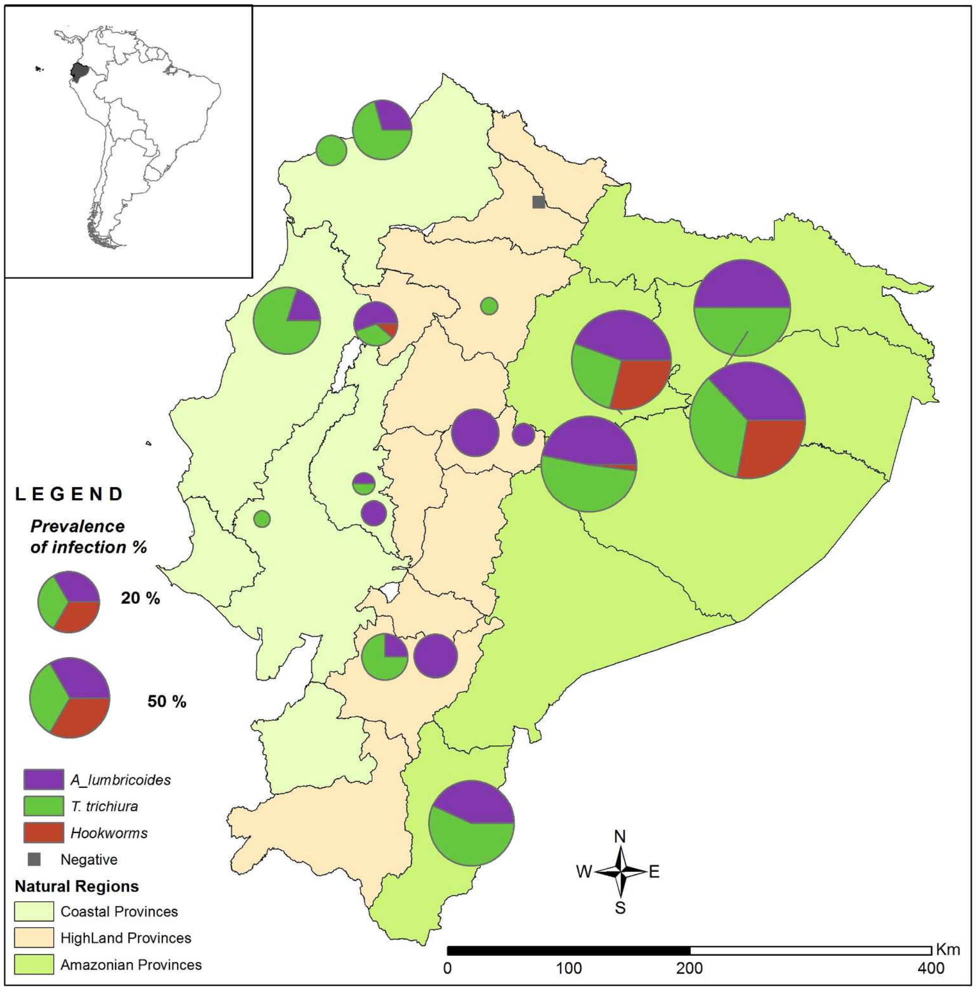

Figure 1

215x219mm (300 x 300 DPI)

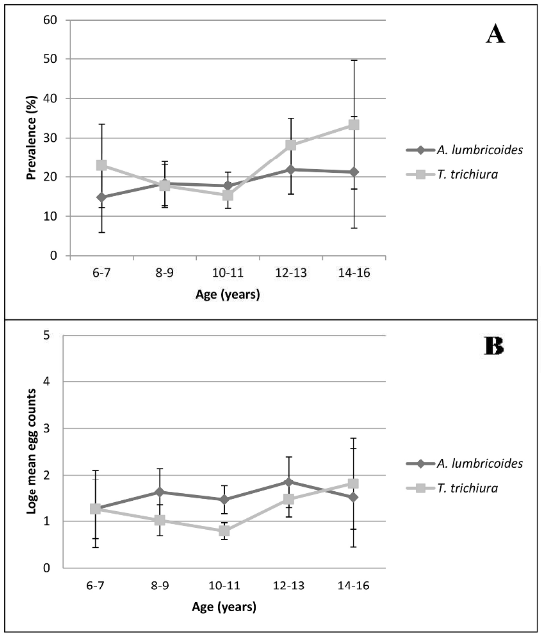

254x190mm (300 x 300 DPI)

STROBE Statement—Checklist of items that should be included in reports of ***cross-sectional studies***

	Item No	Recommendation	Page #
**Title and abstract**	1	(*a*) Indicate the study's design with a commonly used term in the title or the abstract	1
		(*b*) Provide in the abstract an informative and balanced summary of what was done and what was found	2,3
**Introduction**			
Background/rationale	2	Explain the scientific background and rationale for the investigation being reported	4,5
Objectives	3	State specific objectives, including any prespecified hypotheses	5
**Methods**			
Study design	4	Present key elements of study design early in the paper	7
Setting	5	Describe the setting, locations, and relevant dates, including periods of recruitment, exposure, follow-up, and data collection	6,7,8
Participants	6	(*a*) Give the eligibility criteria, and the sources and methods of selection of participants	7
Variables	7	Clearly define all outcomes, exposures, predictors, potential confounders, and effect modifiers. Give diagnostic criteria, if applicable	7,8,9
Data sources/ measurement	8*	For each variable of interest, give sources of data and details of methods of assessment (measurement). Describe comparability of assessment methods if there is more than one group	7,8
Bias	9	Describe any efforts to address potential sources of bias	7,8
Study size	10	Explain how the study size was arrived at	7
Quantitative variables	11	Explain how quantitative variables were handled in the analyses. If applicable, describe which groupings were chosen and why	8
Statistical methods	12	(*a*) Describe all statistical methods, including those used to control for confounding	8,9
		(*b*) Describe any methods used to examine subgroups and interactions	NA
		(*c*) Explain how missing data were addressed	
		(*d*) If applicable, describe analytical methods taking account of sampling strategy	8,9
		(*e*) Describe any sensitivity analyses	NA
**Results**			
Participants	13*	(a) Report numbers of individuals at each stage of study—eg numbers potentially eligible, examined for eligibility, confirmed eligible, included in the study, completing follow-up, and analysed	9
		(b) Give reasons for non-participation at each stage	9
		(c) Consider use of a flow diagram	NA
Descriptive data	14*	(a) Give characteristics of study participants (eg demographic, clinical, social) and information on exposures and potential confounders	9,10
		(b) Indicate number of participants with missing data for each variable of interest	9
Outcome data	15*	Report numbers of outcome events or summary measures	13
Main results	16	(*a*) Give unadjusted estimates and, if applicable, confounder-adjusted estimates and their precision (eg, 95% confidence interval). Make clear	14,15

For peer review only

For peer review only - http://bmjopen.bmj.com/site/about/guidelines.xhtml

		which confounders were adjusted for and why they were included	
		(*b*) Report category boundaries when continuous variables were categorized	11,12
		(*c*) If relevant, consider translating estimates of relative risk into absolute risk for a meaningful time period	NA
Other analyses	17	Report other analyses done—eg analyses of subgroups and interactions, and sensitivity analyses	NA
**Discussion**			
Key results	18	Summarise key results with reference to study objectives	15,16
Limitations	19	Discuss limitations of the study, taking into account sources of potential bias or imprecision. Discuss both direction and magnitude of any potential bias	19,20
Interpretation	20	Give a cautious overall interpretation of results considering objectives, limitations, multiplicity of analyses, results from similar studies, and other relevant evidence	16,17,18,19
Generalisability	21	Discuss the generalisability (external validity) of the study results	17
**Other information**			
Funding	22	Give the source of funding and the role of the funders for the present study and, if applicable, for the original study on which the present article is based	24

*Give information separately for exposed and unexposed groups.

Note: An Explanation and Elaboration article discusses each checklist item and gives methodological background and published examples of transparent reporting. The STROBE checklist is best used in conjunction with this article (freely available on the Web sites of PLoS Medicine at http://www.plosmedicine.org/, Annals of Internal Medicine at http://www.annals.org/, and Epidemiology at http://www.epidem.com/). Information on the STROBE Initiative is available at www.strobe-statement.org.

For peer review only

For peer review only - http://bmjopen.bmj.com/site/about/guidelines.xhtml
